# Supplementary material for: Plasticity in Classical Hodgkin Composite Lymphomas: A Systematic Review
Source: Cancers (Basel). 2022 Nov 19;14(22):5695. doi: 10.3390/cancers14225695 (PMC9688742; doi:10.3390/cancers14225695)
Supplement: Supplementary file 1 [file cancers-14-05695-s001.zip › cancers-1996133-supplementary.pdf]

Supplementary Materials

Supplementary Text S1: classification of treatments.

Supplementary Figure S1: flow diagram.

Supplementary Table S1: PRISMA 2020 checklist.

Supplementary Table S2: clinical and laboratory data of cHL/follicular composite lymphomas.

Supplementary Table S3: histopathological and molecular data of cHL/follicular composite lymphomas.

Supplementary Table S4: clinical and laboratory data data of cHL/mantle-cell composite lymphomas.

Supplementary Table S5: histopathological and molecular data of cHL/mantle-cell composite lymphomas).

Supplementary Table S6: clinical and laboratory data of cHL/marginal zone composite lymphomas.

Supplementary Table S7: histopathological and molecular data of cHL/marginal zone composite lymphomas.

Supplementary Table S8: clinical and laboratory data of cHL/diffuse large B-cells composite lymphomas.

Supplementary Table S9: histopathological and molecular data of cHL/diffuse large B-cells composite lymphomas.

Supplementary Table S10: clinical and laboratory data of cHL/NLPHL composite lymphomas.

Supplementary Table S11: histopathological and molecular data of cHL/NLPHL composite lymphomas.

Supplementary Table S12: clinical and laboratory data of cHL/T-cell composite lymphomas.

Supplementary Table S13: histopathological and molecular data of cHL/T-cell composite lymphomas.

Supplementary Text S1: classification of treatments.

For treatments, since the chemotherapy used were different between studies/case reports, lymphoma treatments were classified in five groups as follows: i) cHL-like chemotherapy; ii) B-cell lymphoma-like chemotherapy (small B-cell and diffuse large B-cell (DLBCL) lymphoma); iii) T-cell lymphoma-like chemotherapy; and iv) composite lymphoma-like chemotherapy (combination of cHL-like chemotherapy and another molecule or chemotherapy to treat both contingents).

cHL-like chemotherapy

ABVD +/- RT (doxorubicin, vinblastine, bleomycin, dacarbazine +/- radiotherapy), ChIVPP (chlorambucil, vinblastine, procarbazine, prednisolone), CVPP (cyclophosphamide, vinblastine, procarbazine, prednisolone), COPP (cyclophosphamide, vincristine, procarbazine, prednisolone), vinblastine (VELBE), IVOx (ifosfamide, etoposide, oxaliplatine), cisplatine + prednisone + BCNU (cyclophosphamide, carmustine), BEACOPP (bleomycin, etoposide, doxorubicin, cyclophosphamide, vincristine, procarbazine, prednisone), BV-ICE (brentuximab, vedotin, ifosfamide, carboplatin, etoposide) were considered as cHL-like chemotherapy.

B-cell lymphoma-like chemotherapy

R-CHOP +/- RT (rituximab, doxorubicin, methylprednisolone, cyclophosphamide, vincristine +/- radiotherapy) or CHOP +/- RT, EPOCH (etoposide, vincristin, adriamycin, cyclophosphamide, prednisone), bendamustine were considered as small B-cell lymphoma-like chemotherapy.

R-CHOP, CHOP, R-DHAOx (rituximab dexamethasone cytarabine oxaliplatin), R-ACVBP (rituximab, doxorubicin, cyclophosphamide, vindesine, bleomycin, prednisone), R-COPADM (rituximab, cyclophosphamide, vincristine, prednisone, adriamycin, high-dose methotrexate) + CYM (cytarabine, methotrexate, prednisone), MACOP-B (methotrexate, doxorubicin, cyclophosphamide, vincristine, prednisone, bleomycin), R-EPOCH, ProMACE-MOPP (prednisone, methotrexate, doxorubicin, cyclophosphamide, etoposide, and mechlorethamine, vincristine, procarbazine, prednisone), ProMACE CytaBOM (cyclophosphamide, doxorubicin, etoposide and cytozar, bleomycin, vincristine, methotrexate prednisone), were classified as DLBCL-like chemotherapy.

T-cell lymphoma-like chemotherapy

CHO(E)P and GDP (Gemcitabine, Dexamethasone, Cisplatin) were considered as T-cell lymphoma-like chemotherapy.

Composite lymphoma-like chemotherapy

The combination of chemotherapies as R-ABVD (rituximab, doxorubicin, vinblastine, bleomycin, dacarbazine), C-MOPP (cyclophosphamide, vincristine, procarbazine, prednisone) + ABVD or MOPP (mechlorethamine, vincristine, procarbazine, prednisone) + ABV, R-BEACOPP, ABVD or CHOP combined with R-ICE (rituximab, ifosfamide, carboplatine, etoposide), ICE + LEED (L-PAM, etoposide, cyclophosphamide, and dexamethazone), ABDV + CHO(E)P or ABDV + chidamide, and RT + R-CODOX-M/IVAC (rituximab, cyclophosphamide, doxorubicin, vincristine, methotrexate/ifosfamide, etoposide, high dose cytarabine), R-DHAOX (rituximab, dexamethasone, high-dose cytarabine, oxaliplatin) were considered as composite lymphoma-like chemotherapy.

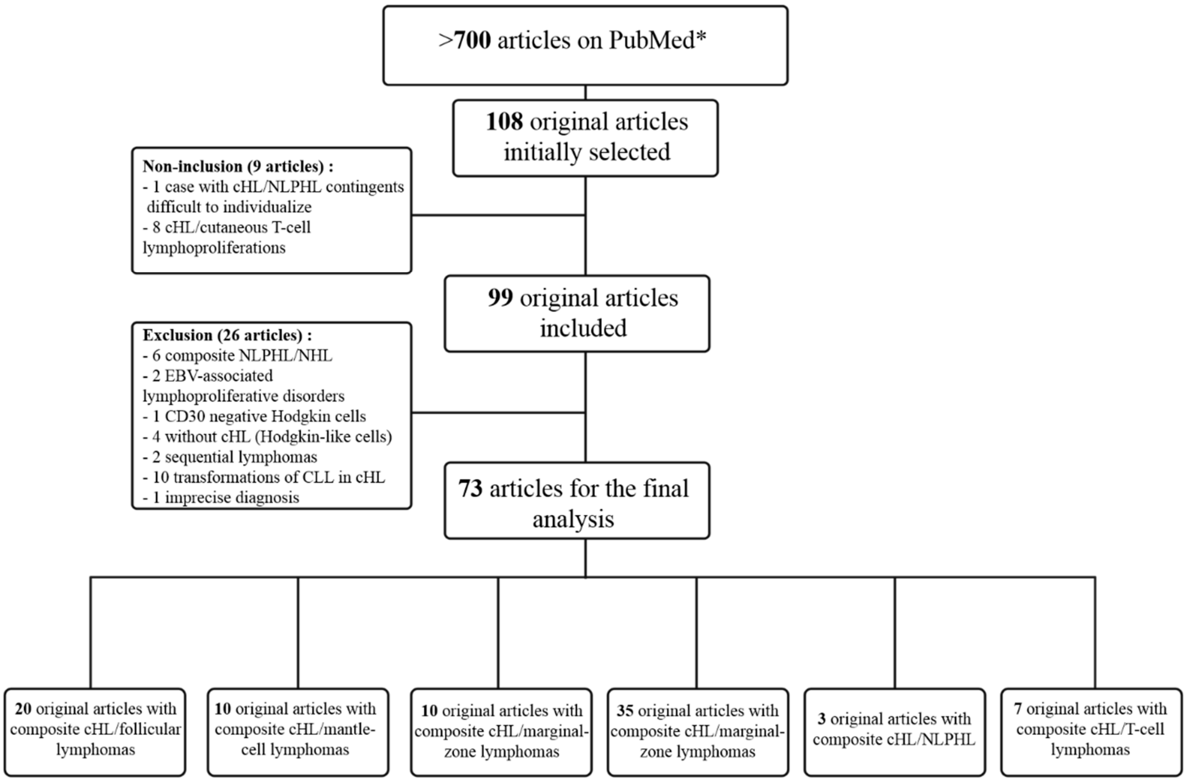

Supplementary Figure S1: flow diagram for original article inclusion/exclusion.

*Among the >700 articles found on PubMed using the keywords "composite lymphoma(s)", "synchronous lymphoma(s)", "composite AND Hodgkin", and "synchronous AND Hodgkin", 108 were initially selected (all composite/synchronous lymphoma case reports or series). However, nine original articles were not included because they did not meet inclusion criteria (see Methods). Among the 99 original articles included, 26 were excluded because they met exclusion criteria (see Methods). As a result, 73 original articles were used for the final analysis. However, many articles reported different types of composite lymphomas, explaining that the sum of all articles is not 73.

CLL: Chronic lymphocytic leukemia; cHL: Classical Hodgkin lymphoma; EBV: Epstein-Barr virus; NHL: Non-Hodgkin lymphoma; NLPHL: Nodular lymphocyte-predominant Hodgkin lymphoma. This figure was created using BioRender (Toronto, Canada).

Supplementary Table S1: PRISMA 2020 checklist.

Section and Topic	Item #	Checklist item	Location where item is reported
**TITLE**			
Title	1	Identify the report as a systematic review.	p. 1
**ABSTRACT**			
Abstract	2	See the PRISMA 2020 for Abstracts checklist.	p. 4
**INTRODUCTION**			
Rationale	3	Describe the rationale for the review in the context of existing knowledge.	p. 5-6
Objectives	4	Provide an explicit statement of the objective(s) or question(s) the review addresses.	p. 5-6
**METHODS**			
Eligibility criteria	5	Specify the inclusion and exclusion criteria for the review and how studies were grouped for the syntheses.	p. 7
Information sources	6	Specify all databases, registers, websites, organisations, reference lists and other sources searched or consulted to identify studies. Specify the date when each source was last searched or consulted.	p. 7
Search strategy	7	Present the full search strategies for all databases, registers and websites, including any filters and limits used.	p. 7
Selection process	8	Specify the methods used to decide whether a study met the inclusion criteria of the review, including how many reviewers screened each record and each report retrieved, whether they worked independently, and if applicable, details of automation tools used in the process.	p. 7
Data collection process	9	Specify the methods used to collect data from reports, including how many reviewers collected data from each report, whether they worked independently, any processes for obtaining or confirming data from study investigators, and if applicable, details of automation tools used in the process.	p. 7
Data items	10a	List and define all outcomes for which data were sought. Specify whether all results that were compatible with each outcome domain in each study were sought (e.g. for all measures, time points, analyses), and if not, the methods used to decide which results to collect.	p. 7, Supp. Material
	10b	List and define all other variables for which data were sought (e.g. participant and intervention characteristics, funding sources). Describe any assumptions made about any missing or unclear information.	p. 7, Supp. Material
Study risk of bias assessment	11	Specify the methods used to assess risk of bias in the included studies, including details of the tool(s) used, how many reviewers assessed each study and whether they worked independently, and if applicable, details of automation tools used in the process.	p. 7
Effect measures	12	Specify for each outcome the effect measure(s) (e.g. risk ratio, mean difference) used in the synthesis or presentation of results.	p. 8
Synthesis methods	13a	Describe the processes used to decide which studies were eligible for each synthesis (e.g. tabulating the study intervention characteristics and comparing against the planned groups for each synthesis (item #5)).	p. 7
	13b	Describe any methods required to prepare the data for presentation or synthesis, such as handling of missing summary statistics, or data conversions.	p. 8
	13c	Describe any methods used to tabulate or visually display results of individual studies and syntheses.	p. 8

Reporting bias assessment	14	Describe any methods used to assess risk of bias due to missing results in a synthesis (arising from reporting biases).	p. 8
Certainty assessment	15	Describe any methods used to assess certainty (or confidence) in the body of evidence for an outcome.	NA
**RESULTS**			
Study selection	16a	Describe the results of the search and selection process, from the number of records identified in the search to the number of studies included in the review, ideally using a flow diagram.	p. 9
	16b	Cite studies that might appear to meet the inclusion criteria, but which were excluded, and explain why they were excluded.	p. 9
Study characteristics	17	Cite each included study and present its characteristics.	p. 9 -17
Risk of bias in studies	18	Present assessments of risk of bias for each included study.	NA
Results of individual studies	19	For all outcomes, present, for each study: (a) summary statistics for each group (where appropriate) and (b) an effect estimate and its precision (e.g. confidence/credible interval), ideally using structured tables or plots.	Tables 1-3
Results of syntheses	20a	For each synthesis, briefly summarise the characteristics and risk of bias among contributing studies.	p. 10-17
	20b	Present results of all statistical syntheses conducted. If meta-analysis was done, present for each the summary estimate and its precision (e.g. confidence/credible interval) and measures of statistical heterogeneity. If comparing groups, describe the direction of the effect.	Table 1-3
	20c	Present results of all investigations of possible causes of heterogeneity among study results.	NA
	20d	Present results of all sensitivity analyses conducted to assess the robustness of the synthesized results.	NA
Reporting biases	21	Present assessments of risk of bias due to missing results (arising from reporting biases) for each synthesis assessed.	NA
Certainty of evidence	22	Present assessments of certainty (or confidence) in the body of evidence for each outcome assessed.	NA
**DISCUSSION**			
Discussion	23a	Provide a general interpretation of the results in the context of other evidence.	p. 18-19
	23b	Discuss any limitations of the evidence included in the review.	p. 24
	23c	Discuss any limitations of the review processes used.	p. 24
	23d	Discuss implications of the results for practice, policy, and future research.	p. 24-25

OTHER INFORMATION			
Registration and protocol	24a	Provide registration information for the review, including register name and registration number, or state that the review was not registered.	p. 8
	24b	Indicate where the review protocol can be accessed, or state that a protocol was not prepared.	NA
	24c	Describe and explain any amendments to information provided at registration or in the protocol.	NA
Support	25	Describe sources of financial or non-financial support for the review, and the role of the funders or sponsors in the review.	p. 28
Competing interests	26	Declare any competing interests of review authors.	p. 28
Availability of data, code and other materials	27	Report which of the following are publicly available and where they can be found: template data collection forms; data extracted from included studies; data used for all analyses; analytic code; any other materials used in the review.	Supp. Material

From: Page MJ, McKenzie JE, Bossuyt PM, Boutron I, Hoffmann TC, Mulrow CD, et al. The PRISMA 2020 statement: an updated guideline for reporting systematic reviews. BMJ 2021;372:n71. doi: 10.1136/bmj.n71

For more information, visit: http://www.prisma-statement.org/

NA: Not assessed.

Supplementary Table S2: clinical and laboratory data of cHL/follicular composite lymphomas.

References	Age (years)	Sex	History of lymphoma	Location	Tumor size (cm)	B symptoms	Ann Arbor stage	Other symptoms	Laboratory data	Extension assessment	Treatment	Outcomes	Follow-up duration (months)	Time of relapse / DOD (months)
Thirumala *et al*., 2000 [15]	73	M	FL (13 years ago, treated by radiation therapy)	Left inguinal area LN	4.5	Yes	I B	Sore throat, and 3-day duration malaise.	NA	CT-scan: no adenopathy nor organomegaly	NA	NA	NA	NA
Urano *et al.*, 2004 [54]	58	M	No	Right parotid gland	2	No	II A	Right parotid swelling since 3 months	NA	CT-scan: no abnormality	CHOP	Complete remission	NA	NA
Vasudevan *et al.*, 2017 (case 1) [55]	48	F	No	Cervical LN	NA	Yes	IV B	NA	NA	NA	R-CHOP + RT	Loss to follow-up	NA	NA
Vasudevan *et al.*, 2017 (case 2) [55]	59	M	No	Cervical LN	NA	No	I A	NA	NA	NA	ABVD + RT	Asymptomatic since 1 year	12	NA
Vasudevan *et al.*, 2017 (case 3) [55]	53	M	No	Cervical LN	NA	No	I A	NA	NA	NA	R-CHOP + RT	Asymptomatic since 11 months	11	NA
Vasudevan *et al.*, 2017 (case4) [55]	44	M	No	Inguinal LN	NA	Yes	II B	NA	NA	NA	R-CHOP	Loss to follow-up	NA	NA
Vasudevan *et al.*, 2017 (case 5) [55]	72	M	No	Perigastric LN	NA	Yes	IV B	NA	NA	NA	No treatment	Loss to follow-up	NA	NA
Huang *et al.*, 2022 (case 1) [56]	75	F	NA	Inguinal LN	NA	NA	NA	NA	NA	NA	NA	DOD	3	3
Huang *et al.*, 2022 (case 2) [56]	64	M	NA	Splenic hilar LN	NA	Yes	IV B	NA	LDH in normal range	BM +: FL, 40%	NA	Alive	57	NA
Huang *et al.*, 2022 (case 3) [56]	57	F	NA	Inguinal LN	NA	No	NA	NA	LDH in normal range	NA	R-ABVD + ASCT	Alive but relapse	17	NA
Huang *et al.*, 2022 (case 4) [56]	51	F	NA	Mesenteric LN	NA	Yes	IV B	NA	LDH in normal range	BM+: FL, 1%	ABVD	Alive	9	NA
Huang *et al.*, 2022 (case5) [56]	82	F	NA	Axillary LN	NA	NA	NA	NA	NA	BM-	NA	NA	NA	NA
Huang *et al.*, 2022 (case 6) [56]	58	F	NA	Paratracheal LN	NA	NA	NA	NA	NA	NA	NA	NA	NA	NA
Huang *et al.*, 2022 (case 7) [56]	46	M	NA	Inguinal LN	NA	Yes	IV B	NA	LDH in normal range	BM+: FL, 5%	MOPP/ABV + ASCT	Alive	82	NA
Huang *et al.*, 2022 (case 8) [56]	50	M	NA	Supraclavicular LN	NA	No	IV A	NA	LDH in normal range	BM+: FL, 5%	ChIVPP/ABV+ASCT	Alive	116	NA
Huang *et al.*, 2022 (case 9) [56]	56	M	NA	Peritracheal LN	NA	NA	NA	NA	NA	NA	NA	NA	NA	NA
Huang *et al.*, 2022 (case 10) [56]	68	F	NA	Cervical LN	NA	NA	NA	NA	LDH in normal range	BM-	NA	DOD	63	63
Huang *et al.*, 2022 (case 11) [56]	43	F	NA	Cervical LN	NA	NA	IV	NA	LDH in normal range	BM+: FL, 5%	R-CHOP	DOD	10	10

Huang *et al.*, 2022 (case 12) [56]	60	M	NA	Supraclavicular LN	NA	NA	NA	NA	NA	NA	NA	NA	NA	NA
Huang *et al.*, 2022 (case 13) [56]	66	M	NA	Axillary LN	NA	Yes	IV B	NA	LDH increase	BM-	NA	DOD	76	76
Huang *et al.*, 2022 (case 14) [56]	57	M	NA	Inguinal LN	NA	No	I A	NA	LDH in normal range	BM-	R-CHOP + RT	Alive, complete remission	107	NA
Huang *et al.*, 2022 (case 15) [56]	67	M	NA	Axillary LN	NA	NA	IV	NA	NA	BM+: FL, 1%	R-ABVD x6, RICE x4	Alive, partial remission	14	NA
Huang *et al.*, 2022 (case 16) [56]	63	M	NA	Inguinal LN	NA	NA	IV	NA	NA	BM+: FL, 10%	NA	NA	NA	NA
Huang *et al.*, 2022 (case 17) [56]	67	M	NA	Cervical LN	NA	NA	NA	NA	NA	NA	NA	NA	NA	NA
Huang *et al.*, 2022 (case 18) [56]	59	F	NA	Cervical LN	NA	No	IV A	NA	LDH in normal range	BM+: FL, 1%	NA	Alive, Partial remission	18	NA
Huang *et al.*, 2022 (case 19) [56]	66	F	NA	Cervical LN	NA	NA	II	NA	LDH in normal range	NA	R-CHOP x6, RICE x2 + RT	Alive, Partial remission	49	NA
Huang *et al.*, 2022 (case 20) [56]	70	F	NA	Epitrochlear LN	NA	NA	I	NA	LDH in normal range	BM-	R-ABVD x4 + RT	Alive, Partial remission	10	NA
Huang *et al.*, 2022 (case 21) [56]	50	M	NA	Mesenteric LN	NA	NA	NA	NA	NA	NA	NA	NA	NA	NA
Huang *et al.*, 2022 (case 22) [56]	63	F	NA	Axillary LN	NA	NA	NA	NA	NA	NA	NA	NA	NA	NA
Trecourt *et al.*, 2022 (case 1) [10]	81	F	No	Axillary, cervical, sub-carina, porto-cava, iliac LN	1	No	III A	No	LDH in normal range, β2microglobulin increase, hypogammaglobulin	PET-scan: Axillary, cervical, sub-carina, porto-cava, iliac LN	VELBE	Alive, Partial remission	6	NA
Trecourt *et al.*, 2022 (case 4) [10]	76	M	No	Parotid gland	3	No	I A	Suspicion of acute parotitis	LDH increase, β2microglobulin in normal range, blood cell count normal	CT-scan: no other lesion	No treatment	NA	24	NA
Trecourt *et al.*, 2022 (case 5) [10]	62	M	No	Mesenteric mass, splenic hilar LN	14	Yes	II B	Dry cough	Anemia, CRP increase	PET-scan: mesenteric mass with splenic hilar LN; BM-	R-ABVD x6	NA	NA	NA
Trecourt *et al.*, 2022 (case 6) [10]	67	M	No	Inguinal LN	7	No	II A	No	LDH, β2microglobulin , and albumin in normal range	CT-scan: iliac and inguinal LN	R-CHOP, then maintenance treatment with R	Complete remission	20	NA
Trecourt *et al.*, 2022 (case 7) [10]	57	F	No	Mesenteric mass	9	Yes	IV B	No	Anemia, LDH and β2microglobulin increase	CT-scan: mesenteric LN; PET-scan: sub- and supra-diaphragmatic lymph nodes	R-ABVD x6	Complete remission	12	NA
Trecourt *et al.*, 2022 (case 8) [10]	79	M	No	Lumbo-aortic LN	NA	Yes	IV B	Hives	NA	CT-scan: mediastinal, retoperitoneal and mesenteric LN; BM+: FL	ABVD x8	Complete remission	84	NA

Trecourt *et al.*, 2022 (case 9) [10]	79	M	No	Inguinal, axillary, lumbo-aortic, retroperitoneal LN	5,3	Yes	III B	NA	Anemia, β2microglobulin increase, LDH in normal range	NA	R-CHOP x6, then MOPP for relapse	Complete remission but relapse after 4 months, and then complete remission	95	4
Trecourt *et al.*, 2022 (case 11) [10]	81	M	No	supraclavicular LN	NA	No	II A	No	NA	CT-scan : retroperitoneal, mesenteric, supraclavicular LN	ABVD x4 + RT	Complete remission, but DOD after 9 years (relapse on FL)	108	108
Trecourt *et al.*, 2022 (case 22) [10]	58	M	No	Cutaneous lesions and deltoid, mediastinal, supraclavicular and cervical LN	7	No	IV A	Pruritus	LDH, β2microglobulin, CRP, and hemoglobin in normal range	NA	R-ABVD x6, for the relapse 8 months later: R-CVP but DOD 10 months after diagnosis of FL	Complete remission during 8 months, and relapse (unspecified)	12	8
Trecourt *et al.*, 2022 (case 3) [10]	81	M	FL	Axillary LN Parotid gland	NA	No	IV A	No	LDH and CRP in normal range	NA	R-CHOP x8	Complete remission	60	NA
Trecourt *et al.*, 2022 (case 10) [10]	58	M	FL	Retroperitoneal LN, cutaneous location	NA	NA	NA	NA	NA	CT-scan: retroperitoneal LN and a sub-cutaneous lesion	R-DHAOX x6, and then MINE-R	Partial remission after first therapy, relapse 11 months later and DOD 15 months after the diagnosis	15	11
Trecourt *et al.*, 2022 (case 12) [10]	56	M	cHL (8 years earlier)	Mediastinum	NA	NA	NA	NA	NA	NA	Brentuximab-Bendamustine, gemcitabine and R-IVA x3	Complete remission and relapse 14 months later and DOD 17 months after the relapse	17	14
Maeshima *et al.*, 2015 (case 1) [3]	40	F	No	Unspecified (LN)	NA	NA	I	NA	NA	NA	C-MOPP, ABVD + RT	Alive, complete remission	171	NA
Maeshima *et al.*, 2015 (case 2) [3]	50	M	No	Unspecified (LN)	NA	NA	IV	NA	NA	BM+: FL	R-ABCD	Alive, complete remission	23	NA
Maeshima *et al.*, 2015 (case 3) [3]	50	M	No	Unspecified (LN) and duodenum	NA	NA	III	NA	NA	NA	R-ABCD	Alive, complete remission, but relapse on FL at 31 months	31	31
Maeshima *et al.*, 2015 (case 4) [3]	61	M	No	Unspecified (LN)	NA	NA	III	NA	NA	NA	ABVD	Alive, complete remission, but relapse on FL at 59 months	59	59
Maeshima *et al.*, 2015 (case 5) [3]	45	F	No	Unspecified (LN)	NA	NA	IV	NA	NA	BM+: cHL	R-CHOP, then treatment of the progressive disease using ABVD	DOD (relapse on cHL)	8	8
Küppers *et al.*, 2001 [12]	43	M	No	Splenic hilar LN	NA	NA	IV	Hemophagocytic syndrome	NA	BM+ (autopsy): FL	No treatment	DOD (hemophagocytic syndrome)	0.75	0.75
Gonzalez *et al.*, 1991 (case 2) [14]	43	M	DLBCL 4 years ago and FL 2 years ago	Inguinal LN	NA	No	NA	NA	NA	BM-	Multiagent chemotherapy + ASCT	DOD 26 months after diagnosis of CL	26	26
Gonzalez *et al.*, 1991 (case 4) [14]	62	F	No	Inguinal LN	NA	No	I	NA	NA	BM-	RT	Alive, complete remission	27	NA

Gonzalez *et al.*, 1991 (case 8) [14]	48	M	FL, 12 years ago	Cervical and inguinal LN	NA	Yes	NA	NA	NA	BM-	RT+ nitrogen mustard; vincristine; prednisone; procarbazine	Relapse 18 months later and DOD at 55 months	55	18
Bräuminger *et al.*, 1999 (case 1) [11]	75	F	No	Retroperitoneal LN	NA	Yes	IV B	NA	NA	BM+: FL;<br>Liver involvement (FL)	CVPP x 8	Alive, but relapse 18 months later (not confirmed by biopsy)	18	18
Linck *et al.*, 2005 [57]	38	M	No	Inguinal and cervical LN	NA	No	IV A	NA	Thrombopenia, β2microglobulin increase, low IgG level	CT-scan: multiple cervical, mediastinal, mesenterial and inguinal LN and splenomegaly;<br>BM+: FL, 40-60%	Polychemotherapy (BEACOPP) + R	Alive, complete remission	28	NA
Jaffe *et al.*, 1992 (case 2) [58]	38	M	No	Cervical LN	NA	NA	NA	NA	NA	NA	NA	NA	NA	NA
Jaffe *et al.*, 1992 (case 5) [58]	84	F	No	Cervical LN	NA	NA	NA	NA	NA	NA	NA	NA	NA	NA
Jaffe *et al.*, 1992 (case 13) [58]	66	F	No	Unspecified (LN)	NA	NA	NA	NA	NA	NA	NA	NA	NA	NA
Jaffe et al., 1992 (case 1) [58]	38	M	FL	Cervical, inguinal and submandibular LN	NA	NA	NA	NA	NA	NA	NA	NA	NA	NA
Hansmann *et al.*, 1989 (case 1.3) [59]	68	M	No	Axillary LN	NA	NA	NA	NA	NA	NA	NA	NA	NA	NA
Hansmann *et al.*, 1989 (case 1.10) [59]	52	F	No	LN (unspecified)	NA	NA	NA	NA	NA	NA	NA	NA	NA	NA
Hansmann *et al.*, 1989 (case 2.1) [59]	68	M	No	LN (unspecified)	NA	NA	NA	NA	NA	NA	NA	NA	NA	NA
Hansmann *et al.*, 1989 (case 2.2) [59]	68	M	No	Cervical LN	NA	NA	NA	NA	NA	NA	NA	NA	NA	NA
Hansmann *et al.*, 1989 (case 2.3) [59]	37	F	No	Supraclavicular LN	NA	NA	NA	NA	NA	NA	NA	NA	NA	NA
Hansmann *et al.*, 1989 (case 2.4) [59]	40	M	No	Cevical LN	NA	NA	NA	NA	NA	NA	NA	NA	NA	NA
Hansmann *et al.*, 1989 (case 2.5) [59]	70	F	No	Unspecified (LN)	NA	NA	NA	NA	NA	NA	NA	NA	NA	NA
Demurtas *et al.*, 2011 (case 16) [60]	79	M	No	Inguinal LN	NA	No	NA	Weight loss	NA	NA	NA	NA	NA	NA
Yoshida *et al.*, 2012 (case 1) [61]	56	F	No	Cervical LN	NA	NA	NA	NA	NA	NA	NA	NA	NA	NA
Yoshida *et al.*, 2012 (case 2) [61]	71	M	No	Pharynx	NA	NA	NA	NA	NA	NA	NA	NA	NA	NA

Wang *et al.*, 2016 (case 1) [62]	63	M	No	Supraclavicular, mediastinal, mesenteric, retroperitoneal LN	2,3	No	III A	Fatigue	NA	NA	R-CHOP x8 (partial remission), then after 1.5 years ABVDx6 then brentuximabx2 but partial remission, ICE (non-responsive)	Alive, with cHL refractory to R-CHOP after 18 months	34	18
Kim *et al.,* 2022 [63]	40	M	No	Neck mass (regions I, II, V), both axillary, and retropancreatic LN	NA	Yes	IV A	NA	NA	CT-scan/PET-scan: no organomegaly but multiple hypermetabolic LN and mild irregular hypermetabolism of the spleen and along the marrox-space.	ABVD then relapse treated by 6x bendamustine + rituximab	Relapse at 6 months on FL (grade 3a)	6	6
Nishioka *et al.,* 2018 [64]	73	F	No	Systemic LN and ulcerative mass of the esophagus	1,5	Yes	IV B	NA	Soluble IL-2R and LDH increase	PET-scan: cervical, axillary, mediastinal, supraclavicular, para-aortic, and inguinal LN Esophagogastroduodenoscopy: ulcerative mass in the mid-esophagus; BM+ (*IgH* rearrangment identified, whitout tumor cell vizualised)	8x R-CHOP, and DeVIC x4 for the relapse	Complete remission, then relapse (1 month later on cHL), then complete remission	1	1
Pezzella *et al.,* 2018 [65]	64	M	No	Ileus	NA	No	IV A	Epigastric/perianal pain and intestinal obstruction	β2microglobulin mildly increase, hemoglobin, platelet, with blood cell, LDH in normal range	Abdominal MRI: ascite and a large bulky mass in the mesenteric region, with thickening and mild dilatation of the involved small bowel walls, no BM involvement on MRI	NA	NA	NA	NA
Schmitz *et al.,* 2005 (case 1) [6]	NA	NA	NA	NA	NA	NA	NA	NA	NA	NA	NA	NA	NA	NA
Schmitz *et al.,* 2005 (case 2) [6]	NA	NA	NA	NA	NA	NA	NA	NA	NA	NA	NA	NA	NA	NA
Kingma *et al.,* 1994 (case 7) [66]	35	M	No	Inguinal LN	NA	NA	NA	NA	NA	NA	NA	NA	NA	NA
Kingma *et al.,* 1994 (case 8) [66]	38	M	No	Cervical LN	NA	NA	NA	NA	NA	NA	NA	NA	NA	NA
Kingma *et al.,* 1994 (case 10) [66]	63	M	no	Unspecified (LN)	NA	NA	NA	NA	NA	NA	NA	NA	NA	NA
Kingma *et al.,* 1994 (case 11) [66]	58	M	no	Cervical LN	NA	NA	NA	NA	NA	NA	NA	NA	NA	NA

ASCT: Autologous stem-cell transplantation; BM+/-: Involvement of bone marrow or not; cHL: classical Hodgkin lymphoma; CRP: C reactive protein; CT-scan: computed tomography-scan; DLBCL: Diffuse large B cell lymphoma; DOD: Dead of disease; F: Female; FL: Follicular lymphoma; IL-2R: Interleukin 2 receptor; LDH: Lactate dehydrogenase; LN: Lymph node(s); M: Male; NA: Not available; PET-scan: Positron emission tomography-scan. For chemotherapy abbreviations, please see **Supplementary Text S1**.

Supplementary Table S3: histopathological and molecular data of cHL/follicular composite lymphomas.

References	cHL type	FL grade	Separated / mixed contingents (*)	Immunophenotype cHL	Immunophenotype FL	*BCL2* /BCL6 translocations (cHL)	*BCL2*/BCL6 translocations (FL)	Clonal B rearrangements (cHL)	Clonal B rearrangements (FL)	Other molecular alterations
Thirumala *et al*., 2000 [15]	cHL-NS	Grade 1-2	Separated	CD30+, CD15+, CD20-	NA	NA	NA	NA	NA	NA
Urano *et al.,* 2004 [54]	cHL-MC	Grade 2	Separated (FL, 90%)	CD30+, CD15+, CD20+, CD79a+, CD10-, CD5-, CyclineD1-, CD45+, BCL2+ (focal), UCHL1-, EBER+ (focal)	CD20+, CD45+, CD79a+, BCL2+, CD10+, CD5-, UCHL1-, Bel1-	NA	NA	NA	NA	On whole tissue sample: t(3;14;18) gene translocation
Vasudevan *et al.,* 2017 (case 1) [55]	cHL-MC	Grade 1-2	Mixed	CD30+, CD15-, CD20-, PAX5+, CD45-	CD20+, BCL6+	NA	NA	NA	NA	NA
Vasudevan *et al.,* 2017 (case 2) [55]	cHL-MC	Grade 1-2	Mixed (interfollicular)	CD30+, CD15-, CD20+, PAX5+, CD45-	CD20+, BCL2+, BCL6+	NA	NA	NA	NA	NA
Vasudevan *et al.,* 2017 (case 3) [55]	cHL-MC	Grade 1-2	Mixed (interfollicular)	CD30+, CD15+ (focal), CD20-, PAX5+, CD45-	CD20+, BCL2+, BCL6+	NA	NA	NA	NA	NA
Vasudevan *et al.,* 2017 (case4) [55]	cHL-MC	Grade 1-2	Mixed (interfollicular)	CD30+, CD15-, CD20-, PAX5+, CD45-	CD20+, BCL6+, BCL2+	NA	NA	NA	NA	NA
Vasudevan *et al.,* 2017 (case 5) [55]	cHL-MC	Grade 1-2	Mixed (interfollicular)	CD30+, CD15+ (focal), CD20-, PAX5+, CD45-	CD20+, BCL6+, BCL2+	NA	NA	NA	NA	NA
Huang *et al.,* 2022 (case 1) [56]	cHL-MC	Grade 2	Mixed (interfollicular), equal quantity	CD30+, CD15+, CD20-, PAX5+, CD10-, CD45-, BCL6-, BCL2-, EBER+	CD20+, CD45+, BCL6+, BCL2+, CD10+, EBER-	NA	NA	NA	NA	NA
Huang *et al.,* 2022 (case 2) [56]	cHL-MC	Grade 3A	Separated (FL, 70%)	CD30+, CD15+, CD20-, PAX5+, CD45-, BCL2-, BCL6-, EBER-	CD20+, CD45+, BCL2-, BCL6+, EBER-	NA	NA	NA	NA	On whole tissue sample: clonal *IgH* rearrangement
Huang *et al.,* 2022 (case 3) [56]	cHL-MC	Grade 2	Separated (FL, 65%)	CD30+, CD15-, CD20-, PAX5+, CD10-, CD45-, BCL6+, BCL2+, EBER-	CD20+, CD45+, BCL2+, BCL6+, CD10+, EBER-	NA	*BCL2* translocation	NA	NA	NA
Huang *et al.,* 2022 (case 4) [56]	cHL-NS	Grade 1	Separated (HL, 60%)	CD30+, CD15+, CD20+ (focal), PAX5+, CD10-, CD45-, BCL2-, BCL6-, EBER-	CD20+, CD45+, BCL2+, BCL6+, CD10+, EBER-	Same *BCL2* translocation	Same *BCL2* translocation	NA	NA	On whole tissue sample: clonal *IgH* rearrangement
Huang *et al.,* 2022 (case5) [56]	cHL-MC	Grade 3B	Separated (FL, 70%)	CD30+, CD15+, CD20+ (focal), PAX5+, CD10-, CD45-, BCL2+, BCL6+, EBER-	CD20+, CD45+, BCL2+, BCL6+, CD10+, EBER-	NA	*BCL2* translocation	NA	NA	NA
Huang *et al.,* 2022 (case 6) [56]	cHL-MC	Grade 1	Separated (FL, 60%)	CD30+, CD15+, CD20+ (focal), PAX5+, CD10-, CD45-, BCL2-, BCL6-	CD20+, CD45+, BCL2+, BCL6+, CD10+	NA	NA	NA	NA	NA
Huang *et al.,* 2022 (case 7) [56]	cHL-NS	Grade 1	Separated (FL, 80%)	CD30+, CD15-, CD20-, PAX5+, CD45-	CD20+, CD45+	NA	NA	NA	NA	NA

Huang *et al.,* 2022 (case 8) [56]	cHL-MC	Grade 2	Separated (FL, 90%)	CD30+, CD15+, CD20+ (focal), PAX5+, CD45+ (focal), BCL2-, BCL6-	CD20+, CD45+BCL2+, BCL6+	NA	NA	NA	NA	NA
Huang *et al.,* 2022 (case 9) [56]	cHL-NS	Grade 3B	Separated, equal quantity	CD30+, CD15-, CD20-, PAX5+, CD10-, CD45-, BCL2-, BCL6-, EBER-	CD20+, CD45+, BCL2+, BCL6+CD10-, EBER-	NA	NA	NA	NA	NA
Huang *et al.,* 2022 (case 10) [56]	cHL-MC	Grade 2	Separated (FL, 95%)	CD30+, CD15+, CD20+ (focal), PAX5+CD10-, CD45-, BCL2-, EBER+	CD20+, CD45+BCL2+, BCL6+CD10+, EBER-	NA	*BCL2* translocation	NA	NA	NA
Huang *et al.,* 2022 (case 11) [56]	cHL-MC	Grade 2	Separated (FL, 95%)	CD30+, CD15-, CD20+ (focal), PAX5+, CD10-, CD45-, BCL2+, BCL6+, EBER-	CD20+, CD45+, BCL2+, BCL6+, CD10-, EBER-	NA	NA	NA	NA	NA
Huang *et al.,* 2022 (case 12) [56]	cHL-MC	Grade 2	Separated (HL, 60%)	CD30+, CD15-, CD20-, PAX5+, CD10-, EBER-	CD20+, CD45+BCL2+, CD10+	NA	NA	NA	NA	NA
Huang *et al.,* 2022 (case 13) [56]	cHL-MC	Grade 3A	Separated (HL, 80%)	CD30+, CD15-, CD20+ (focal), PAX5+, CD45-, BCL6+, EBER+	CD20+, CD45+BCL2+, BCL6+, EBER-	NA	NA	NA	NA	NA
Huang *et al.,* 2022 (case 14) [56]	cHL-MC	Grade 3A	Separated (FL, 70%)	CD30+, CD15+, CD20-, PAX5+, CD45-	CD20+, CD45+, BCL2+, BCL6+, CD10+	NA	NA	NA	NA	NA
Huang *et al.,* 2022 (case 15) [56]	cHL-MC	Grade 1	Separated (HL, 60%)	CD30+, CD15-, CD20-, PAX5+, CD10-, CD45-, BCL2-, BCL6-, EBER-	CD20+, CD45+, BCL2+, BCL6+, CD10+, EBER-	NA	NA	NA	NA	NA
Huang *et al.,* 2022 (case 16) [56]	cHL-MC	Grade 3A	Separated (FL, 90%)	CD30+, CD15-, CD20+ (weak), PAX5+, CD10-, CD45-	CD20+, CD45+, CD10-	NA	NA	NA	NA	NA
Huang *et al.,* 2022 (case 17) [56]	cHL-MC	Grade 1	Separated (FL, 70%)	CD30+, CD15+, CD20-, PAX5+, CD10-, BCL6+, EBER+	CD20+, CD45+, BCL2+, BCL6+, CD10+, EBER-	NA	NA	NA	NA	NA
Huang *et al.,* 2022 (case 18) [56]	cHL-MC	Grade 3A	Separated (FL, 90%)	CD30+, CD20-, PAX5+, CD45-, BCL6+	CD20+, CD45+BCL2+, BCL6+, CD10+	NA	NA	NA	NA	NA
Huang *et al.,* 2022 (case 19) [56]	cHL-MC	Grade 3A	Separated (FL, 60%)	CD30+, CD15+, CD20-, PAX5+, CD45-, BCL6-, EBER-	CD20+, CD45+, BCL2+, BCL6+, CD10+, EBER-	NA	NA	NA	NA	NA
Huang *et al.,* 2022 (case 20) [56]	cHL-MC	Grade 3A	Separated (HL, 60%)	CD30+, CD15+, CD20-, PAX5+, CD10-, CD45-, BCL2-, BCL6-, EBER-	CD20+, CD45+, BCL6+, BCL2+, CD10+, EBER-	NA	NA	NA	NA	NA

Huang *et al.*, 2022 (case 21) [56]	cHL-MC	Grade 1	Separated (FL, 60%)	CD30+, CD15+, CD20-, PAX5+, CD10-, CD45-, BCL2+, BCL6+, EBER-	CD20+, CD45+, BCL2+, BCL6+, CD10+, EBER-	Same *BCL2* translocation	Same *BCL2* translocation	NA	NA	NA
Huang *et al.*, 2022 (case 22) [56]	cHL-NS	Grade 3A	Separated (HL, 90%)	CD30+, CD15+, CD20-, PAX5+, CD10-, CD45-, BCL2-, BCL6-, EBER-	CD20+, CD45+, BCL2+, BCL6+, CD10+, EBER-			NA	NA	Common 16p duplication
Trecourt *et al.*, 2022 (case 1) [10]	G	Grade 1-2	Separated (FL)	CD30+, CD15+ (focal), CD20-, PAX5+, CD10-, BCL2-, BCL6-, EBER+, MUM1+	CD20-+, BCL2+, BCL6+, CD10+, CD30-, CD15- EBER-, MUM1-, PAX5+	Same *BCL2* translocation; No *BCL6* translocation	Same *BCL2* translocation; No *BCL6* translocation	NA	NA	Common variants of *BCL2, EP300, BCOR;* cHL only: variants of *XPO1, TNFAIP3;* FL only: variants of *BCL2, KMT2D, EP300*
Trecourt *et al.*, 2022 (case 4) [10]	G	Grade 3A	Separated (FL)	CD30+, CD15-, CD20-, PAX5-, CD10-, BCL2-, BCL6-, EBER+, BOB1+, OCT2-, MUM1-	CD20+, BCL2+, BCL6+, CD10-, CD30-, CD15-, EBER-, MUM1-, PAX5+, BOB1-, OCT2+	No *BCL6* translocation	*BCL2* translocation; No *BCL6* translocation	NA	NA	NA
Trecourt *et al.*, 2022 (case 5) [10]	G	Grade 1-2	Separated (HL)	CD30+, CD15-, CD20-, PAX5+, CD79a+ (focal), CD10-, BCL2-, BCL6-, EBER-, BOB1+, OCT2+ (focal), MUM1+	CD20+, CD79a+, BCL2+, BCL6+CD10+, CD30-, CD15-, EBER-, MUM1-, PAX5+, BOB1-, OCT2-	Same *BCL2* translocation; No *BCL6* translocation	Same *BCL2* translocation; No *BCL6* translocation	NA	NA	Common variants of *ARID1A, EP300, KMT2D, SF3B1*; cHL only: variants of *CREBBP* FL only: variants of *BCL2, KMT2D*
Trecourt *et al.*, 2022 (case 6) [10]	G	Grade 1-2	Mixed (FL)	CD30+, CD15-, CD20+ (focal), PAX5+, CD10-, BCL6-, EBER+, BOB1+, OCT2+, MUM1+	CD20+, BCL2-, BCL6+, CD10+, CD30-, CD15-, EBER-, MUM1-	No *BCL2/BCL6* translocation	No *BCL2/BCL6* translocation	NA	NA	NA
Trecourt *et al.*, 2022 (case 7) [10]	G	Grade 1-2	Mixed (FL)	CD30+, CD15+ (focal), CD20-, PAX5+, CD10-, BCL6-, BCL2-, EBER-	CD20+, BCL2+, BCL6+, CD10+, CD30-, CD15-, EBER-, PAX5+	NA	No *BCL2/BCL6* translocation	NA	NA	NA
Trecourt *et al.*, 2022 (case 8) [10]	cHL-NS	Grade 1-2	Separated (synchronous)	CD30+, CD15+, CD20-, PAX5+, CD79a-, CD10+, BCL2-, BCL6+, EBER-, BOB1-, OCT2-, MUM1+	CD20+, BCL2+, BCL6+, CD10+, CD30-, EBER-	No *BCL2/BCL6* translocation	No *BCL2/BCL6* translocation	Common *IgH* rearrangement	Common *IgH* rearrangement	NA
Trecourt *et al.*, 2022 (case 9) [10]	cHL-NS	Grade 1-2	Mixed	CD30+, CD15+ (focal), CD20-, PAX5+, CD79a-, CD10-, BCL2+, BCL6+, EBER+, BOB1-, OCT2-, MUM1+	CD20+, CD79a+, BCL2+, BCL6+, CD10+, CD30-, CD15-, EBER-, MUM1-, PAX5+, BOB1+, OCT2+	Same *BCL2* translocation; No *BCL6* translocation	Same *BCL2* translocation; No *BCL6* translocation	NA	NA	NA

Trecourt *et al.*, 2022 (case 11) [10]	cHL-NS	Grade 1-2	Mixed (HL)	CD30+, CD15-, CD20-, PAX5+, CD79a-, CD10-, BCL2-, BCL6-, EBER-, BOB1-, OCT2-, MUM1+	CD20+, CD79a+, BCL2(E17)+, BCL6+, CD10+, CD30-, CD15-, EBER-, MUM1+, PAX5+	Same *BCL2/BCL6* translocation	Same *BCL2/BCL6* translocation	Common *IgH* rearrangement	Common *IgH* rearrangement	NA
Trecourt *et al.*, 2022 (case 22) [10]	G	Grade 1-2	Mixed (HL)	CD30+, CD15+, CD20-, PAX5+, CD79a-, CD10-, BCL2+, BCL6-, EBER-, BOB1-, OCT2-, MUM1+	CD20+, BCL2+, BCL6+, CD10+ (focal), CD30-, CD15-, EBER-, MUM1-, PAX5+, BOB1+, OCT2+	No *BCL2/BCL6* translocation	No *BCL2/BCL6* translocation	DNA degradation	Clonal *IgH/IgK* rearrangement	NA
Trecourt *et al.*, 2022 (case 3) [10]	G	Grade 3A	Separated	CD30+, CD15-, CD20+ (focal), CD79a+ (focal), CD10+ (focal), BCL2+, BCL6+ (focal), EBER+, MUM1+	CD20+, CD79a-, BCL2+, BCL6+, CD10+, CD30-, CD15-, EBER-, MUM1-	Same *BCL2* translocation; No *BCL6* translocation	Same *BCL2* translocation; No *BCL6* translocation	NA	NA	NA
Trecourt *et al.*, 2022 (case 10) [10]	cHL-NS	Grade 1-2	Separated (synchronous)	CD30+, CD15-, CD20-, CD79a-, PAX5+, BOB1+, OCT2+, MUM1+, CD10-, BCL6+, BCL2-, EBER-	CD20+, CD79a+, CD30-, CD10+, BCL6+, BCL2+, EBER-	Same *BCL2* translocation; No *BCL6* translocation	Same *BCL2* translocation; No *BCL6* translocation	Common *IgH* rearrangement	Common *IgH* rearrangement	NA
Trecourt *et al.*, 2022 (case 12) [10]	G	Grade 1-2	Mixed	CD30+, CD15+, CD20-, CD79a-, PAX5+, BOB1+, OCT2+, MUM1+, CD10-, BCL6+, BCL2+, EBER-	CD20+ (weak), CD79a+, PAX5+, BOB1+, OCT2+, CD30-, CD15-, MUM1+, CD10+, BCL6+, BCL2+, EBER-	No *BCL2/BCL6* translocation	No *BCL2/BCL6* translocation	NA	NA	NA
Maeshima *et al.*, 2015 (case 1) [3]	cHL	Grade 2	Mixed (intefollicular)	CD30+, CD15-, CD20+ (focal), PAX5+, CD79a-, EBER-, BOB1-, OCT2+	CD20+, BCL2+, CD10+, CD30-	Same *BCL2* translocation	Same *BCL2* translocation	NA	NA	NA
Maeshima *et al.*, 2015 (case 2) [3]	cHL-MC	Grade 1	Separated (synchronous)	CD30+, CD15+, CD20-, PAX5+, CD79a+ (focal), EBER-, BOB1-, OCT2-	CD20+, BCL2+, CD10+, CD4-	Same *BCL2* translocation	Same *BCL2* translocation	NA	NA	NA
Maeshima *et al.*, 2015 (case 3) [3]	cHL-MC	Grade 1	Separated (synchronous)	CD30+, CD15-, CD20-, PAX5+CD79a+ (focal), EBER-, BOB1-, OCT2-	CD20+, BCL2+, CD10+	Same *BCL2* translocation	Same *BCL2* translocation	NA	NA	NA
Maeshima *et al.*, 2015 (case 4) [3]	cHL-MC	Grade 2	separated (synchronous)	CD30+, CD10+, CD15+, CD20+ (focal), PAX5+CD79a-, EBER-	CD20+, BCL2+, CD10+	No *BCL2* translocation	NA	NA	NA	NA
Maeshima *et al.*, 2015 (case 5) [3]	cHL	Grade 1	Separated (synchronous)	CD30+, CD15+, CD20+ (focal), PAX5+, CD79a+ (focal), EBER-,	CD20+, BCL2+CD10+	NA	*BCL2* translocation	NA	NA	NA

				BOB1+ (focal), OCT2+ (focal)						
Küppers *et al.,* 2001 [12]	cHL-MC	NA	NA	CD30+, CD15+, CD20-, EBER-	CD20+, CD10+, CD30-, CD15-, EBER-	NA	NA	Common *IgH/IgK* rearrangements, common and different *V* gene somatic hypermutations	Common *IgH/IgK* rearrangements, common and different *V* gene somatic hypermutations	NA
Gonzalez *et al.,* 1991 (case 2) [14]	cHL-NS	NA	NA (NHL, 75-90%)	CD15+, CD20+, CD45-, UCHL1-	CD20+, CD45+, UCHL1-, CD15-	NA	NA	NA	NA	NA
Gonzalez *et al.,* 1991 (case 4) [14]	cHL-NS	NA	NA, equal quantity	NA	NA	NA	NA	NA	NA	NA
Gonzalez *et al.,* 1991 (case 8) [14]	cHL-NS	NA	NA (NHL, 90%)	CD15+, CD20-	CD20+, CD45+, CD15-	NA	NA	NA	NA	NA
Bräuminger *et al.,* (case 1) 1999 [11]	cHL-MC	Grade 1-2	Mixed	CD30+, CD15+, CD20+ (focal)	CD20+, CD30-, CD15-	NA	NA	Common *IgH/IgK* rearrangements, common and different *V* gene somatic hypermutations	Common *IgH/IgK* rearrangements, common and different *V* gene somatic hypermutations	NA
Linck *et al.,* 2005 [57]	cHL-MC	Grade 1-2	NA	CD30+, CD15-, CD20+, BCL2+	CD20+, BCL2+, CD10+	NA	NA	NA	NA	NA
Jaffe *et al.,* 1992 (case 2) [58]	cHL	NA	NA	CD15+, CD20-	NA	NA	NA	NA	NA	NA
Jaffe *et al.,* 1992 (case 5) [58]	cHL	NA	NA	CD15+, CD20-	NA	NA	NA	NA	NA	NA
Jaffe *et al.,* 1992 (case 13) [58]	cHL-NS	NA	NA	CD15+, CD20-	NA	NA	NA	NA	NA	NA
Jaffe *et al.,* 1992 (case 1) [58]	cHL-NS	NA	NA	CD15+, CD20-	NA	NA	NA	NA	NA	NA
Hansmann *et al.,* 1989 (case 1.3) [59]	cHL-MC	NA	NA	CD30+, CD15+	CD15-	NA	NA	NA	NA	NA
Hansmann *et al.,* 1989 (case 1.10) [59]	cHL-NS	NA	NA	CD30+, CD15+	CD15-	NA	NA	NA	NA	NA
Hansmann *et al.,* 1989 (case 2.1) [59]	cHL-MC	NA	NA	CD30+, CD15-	NA	NA	NA	NA	NA	NA
Hansmann *et al.,* 1989 (case 2.2) [59]	cHL-MC	NA	NA	CD15-	NA	NA	NA	NA	NA	NA
Hansmann *et al.,* 1989 (case 2.3) [59]	cHL-MC	NA	NA	CD30+, CD15-	NA	NA	NA	NA	NA	NA
Hansmann *et al.,* 1989 (case 2.4) [59]	cHL-MC	NA	NA	CD15-	NA	NA	NA	NA	NA	NA
Hansmann *et al.,* 1989 (case 2.5) [59]	cHL-NS	NA	NA	CD15-	NA	NA	NA	NA	NA	NA
Demurtas *et al.,* 2011 (case 16) [60]	cHL	Grade 1-2	NA	NA	CD10+ (cytometry)	NA	NA	NA	NA	On whole tissue simple: *BCL2* translocation, no clonal B rearrangement,
Yoshida *et al.,* 2012 (case 1) [61]	cHL-MC	FL *in situ*	NA	CD30+, CD15+, CD20-, BCL2+, EBER+ (focal)	CD20+, BCL2+, CD3-, CD30-, CD15-	Same *BCL2* translocation	Same *BCL2* translocation	NA	NA	cHL contingent: 55,XX,+add(3)(q11.2),-4,-6,add(7)(q11.2), -8,-9,-11,-13,-13,?t(14;18)(q32;q21),

										add(15)(p11.2),+15 mar [1/4]
Yoshida *et al.,* 2012 (case 2) [61]	cHL-MC	Grade 2	NA	CD30+, CD15+, CD20-, BCL2+	CD20+, BCL2+, CD3-, CD30-, CD15-	Not interpretable	*BCL2* translocation	NA	NA	NA
Wang *et al.,* 2016 (case 1) [62]	cHL-MC	Grade 1-2	Separated (synchronous)	CD30+, CD15+, CD20+ (focal), PAX5+, CD79a+ (focal), CD10-, CD5+, CD3+ (focal), CD45+ (focal), BCL2+, EBER-, MUM1+	CD20+, CD45+, BCL2+, CD10+, CD3-, CD15-, EBER-	Same *BCL2* translocation	Same *BCL2* translocation	Common *IgH* rearrangement	Common *IgH* rearrangement	NA
Kim *et al.,* 2022 [63]	cHL-NS	Grade 1	Separated	CD30+, CD15+, CD20-, PAX5+, CD3-, EBER-, MUM1+	CD20+, BCL2+, CD10+, CD3-, CD15-, EBER-	NA	NA	NA	NA	NA
Nishioka *et al.,* 2018 [64]	cHL	NA	Mixed (interfollicular areas)	CD30+, CD15+, EBER-	CD20+, BCL2+ (weak), CD10+, CD3-	NA	NA	Common *IgH* rearrangement	Common *IgH* rearrangement	On whole tissue sample: complex karyotype t(14:18)(q32:p21), and t(14:18) (*BCL2/IgH* translocation)
Pezzella *et al.,* 2018 [65]	cHL	Grade 1-2	NA (FL, 90%)	CD30+, CD20+ (focal), PAX5+, CD10-, CyclinD1-, BCL2+, BCL6-, EBER-, MUM1+	CD20+, BCL2+, BCL6+ (weak), CyclinD1-, CD3-, CD30-, CD15-, EBER-	NA	NA	NA	NA	NA
Schmitz *et al.,* 2005 (case 1) [6]	cHL	NA	NA	BCL2+, EBER-	EBER-	Same *BCL2* translocation	Same *BCL2* translocation	Common *IgH* rearrangement, common and different *V* gene somatic hypermutations	Common *IgH* rearrangement, common and different *V* gene somatic hypermutations	NA
Schmitz *et al.,* 2005 (case 2) [6]	cHL	NA	NA	BCL2+, EBER-	EBER-	Same *BCL2* translocation	Same *BCL2* translocation	Common *IgH* rearrangement, common and different *V* gene somatic hypermutations	Common *IgH* rearrangement, common and different *V* gene somatic hypermutations	NA
Kingma *et al.,* 1994 (case 7) [66]	cHL-NS	NA	NA	NA	NA	NA	NA	NA	NA	NA
Kingma *et al.,* 1994 (case 8) [66]	cHL	NA	NA	NA	NA	NA	NA	NA	NA	NA
Kingma *et al.,* 1994 (case 10) [66]	cHL	NA	NA	NA	NA	NA	NA	NA	NA	NA
Kingma *et al.,* 1994 (case 11) [66]	cHL	NA	NA	NA	NA	NA	NA	NA	NA	NA

cHL: classical Hodgkin lymphoma; EBER: Epstein-Barr virus-encoded small RNA *in situ* hybridization; FL: Follicular lymphoma; G: Granulomatous; MC: Mixed cellularity; NS: Nodular sclerosis; NA: Not available; NHL: Non-Hodgkin lymphoma; +: positive; -: negative.

*Major/predominant contingent and percentage.

Supplementary Table S4: clinical and laboratory data of cHL/mantle-cell composite lymphomas.

References	Age (years)	Sex	History of lymphoma	Location	Tumor size (cm)	B symptoms	Ann Arbor stage	Other symptoms	Laboratory data	Extension assessment	Treatment	Outcome	Follow-up duration (months)	Time of relapse / DOD (months)
Caleo *et al.*, 2003 (case 1) [67]	61	M	Chronic lymphoproliferative disorder, unspecified 10 years earlier	Splenomegaly whithout peripheral LN	NA	No	IV A	Abdominal pain and distension	Lymphocytosis	NA	NA	NA	NA	NA
Caleo *et al.*, 2003 (case 2) [67]	66	M	No	Tonsillar and eyelid mass and cervical LN	NA	No	II A	No	NA	NA	NA	NA	NA	NA
Giua *et al.*, 2015 [68]	89	F	No	Mediastinal and axillary LN	1.5-3	No	IV A	Pulmonary embolism, splenomegaly, hepatomegaly, syncope	Anemia	CT-scan: axillary and mediastinal LN	Prednisone only	NA	NA	NA
Hayes *et al.*, 2006 [69]	69	F	No	Cervical and bilateral axillary LN	1.6	Yes	VI B	NA	Lymphocytosis, LDH and Ig in normal range	BM+: MCL without cHL	CHOPx3 then fludarabine and cyclophosphamide x3	Partial remission, death of pneumonia 11 months later	11	NA
Kanai *et al.*, 2021 [70]	70	M	MCL 9 years earlier treated by autologous stem cell transplant	Cervical and paraaortic LN, splenomegaly	NA	No	VI A	NA	Anemia, ASAT, ALAT, LDH, Alkaline Phosphatase, bilirubine and soluble interleukin-2 receptor increase	Autopsy: BM+: MCL and HL, hemophagocytosis, bipleen and bile duct CT-scan: cervical and para-aortic LN, splenomegaly	Bendamustine	DOD 54 days after admission (disease progression)	NA	NA
Schmitz *et al.*, 2005 (case 5) [6]	NA	NA	MCL	NA	NA	NA	NA	NA	NA	NA	NA	NA	NA	NA
Kramer *et al.*, 2019 [71]	63	M	Orbital MCL lymphoma 8 years earlier	Cervical and axillary and inguinal LN	NA	No	III A	Facial swelling for 2 months (bilateral parotid gland enlargement)	NA	NA	Bendamustine and Rituximabx6	Partial remission	NA	NA
Murray *et al.*, 2017 [72]	78	M	No	Splenomegaly and neck, supraclavicular, axillary, mediastinal, retroperitoneal LN	NA	No	IV A	Weakness, malaise, dyspnea	LDH increase	PET-scan: splenomegaly and neck, supraclavicular, axillary, mediastinal, retroperitoneal LN and mildly activity of bone marrow;<br>BM+: MCL	R-CHOP-like chemotherapy (rituximab-cytosine-arabinoside alterning with 1 course of high-dose methotrexate)	Complete remission	NA	NA
Schneider *et al.,* 2014 [8]	70	M	MCL	Cervical, axyllary and abdominal LN	NA	No	III A	NA	NA	NA	Vinblastine-adriamycine-decarbamazine x3 alternating with 2x R-CHOP	Clinical remission, but death of sepsis just after treatment	NA	NA
Sharma *et al.*, 2019 [73]	62	M	No	Tonsil and LN	6.4	No	II A	Difficulty swallowing, weight loss	No abnormalities of routine biochemical test and blood count	NA	NA	NA	NA	NA
Tinguely *et al.*, 2003 [9]	42	M	MCL 3 years earlier	Abdominal LN and spleen	NA	NA	II A	NA	NA	NA	NA	NA	NA	NA

ALAT: Alanine-aminotransferase; ASAT: Aspartate-aminotransferase; BM+/-: Involvement or not of bone marrow; cHL: classical Hodgkin lymphoma; CT-scan: computed tomography-scan; DOD: Dead of disease; F: Female; Ig: Immunoglobulin; LDH: Lactate dehydrogenase; LN: Lymph node(s); M: Male; MCL: Mantle-cell lymphoma; NA: Not available; PET-scan: Positron emission tomography-scan. For chemotherapy abbreviations, please see **Supplementary Text S1**.

Supplementary Table S5: histopathological and molecular data of cHL/mantle-cell composite lymphomas.

References	cHL type	MCL type	Separated / mixed contingents	Immunophenotype cHL	Immunophenotype MCL	*CCDN1* translocations (cHL)	*CCDN1* translocations (MCL)	Clonal B rearrangements (cHL)	Clonal B rearrangements (MCL)	Other molecular alterations
Caleo *et al.*, 2003 (case 1) [67]	cHL	Classical	Mixed	CD30+, CD15+, CD20-, CD10-, CD5-, CyclinD1-, IgD-, BCL2-, BCL6-, EBER+, LMP1+, CD23-, CD43-	CD20+, BCL2+, BCL6-, CyclinD1+, IgD+, CD10-, CD43+, CD5-, CD30-, CD15-, EBER-, LMP1-	-	+	Distinct *IgH* rearrangement	Distinct *IgH* rearrangement	cHL: aneuploid; MCL: diploid
Caleo *et al.*, 2003 (case 2) [67]	cHL	Classical	NA	CD30+, CD15+, CD20-, CD10-, CD5-, CD3-, CyclinD1-, IgD-, BCL2-, BCL6-, EBER+, LMP1+, CD23-, CD43-	CD20+, BCL2+, BCL6-, CyclinD1+, IgD+, CD10-, CD5+ (focal), CD23-, CD43+, CD30, CD15-, EBER-, LMP1-	-	+	Distinct *IgH* rearrangement	Distinct *IgH* rearrangement	cHL: aneuploid; MCL: diploid
Giua *et al.*, 2015 [68]	cHL	Classical	NA	CD30+, CD15+, CD20-, CyclinD1-	CD20+, CD79a+, CyclinD1+, CD5-, CD23-	-	+	NA	NA	NA
Hayes *et al.*, 2006 [69]	cHL-NS	Classical	NA	CD30+, CD15+, CD20-, ALK1-, EBER-	CD20+, CD79a+, CyclinD1+, CD5+, CD23+, CD3+, EBER-	NA	NA	NA	NA	On whole tissue sample: t(11;14)
Kanai *et al.*, 2021 [70]	cHL-LR	Classical	Separated	CD30+, CD15+, CD20-, PAX5+, CD5-, CyclinD1+ (focal), SOX11-, EBER+, p53+, BOB1-, OCT2-	CD20+, BCL2+, BOB1-, CyclinD1+, SOX11+, CD10-, CD5-, CD23-, p53+, CD3-, EBER-, PAX5+	Same rearrangement	Same rearrangement	NA	NA	On whole tissue sample: Clonal *IgH* rearrangement
Schmitz *et al.*, 2005 (case 5) [6]	cHL	Classical	NA	BCL2-, EBER+	BCL2+	Same rearrangement	Same rearrangement	Distinct *IgH* rearrangement, presence of *V* gene somatic hypermutations	Distinct *IgH* rearrangement, absence of *V* gene somatic hypermutations	No *TP53* mutation (both contingents)
Kramer *et al.*, 2019 [71]	cHL	Classical	Mixed	CD30+, CD15+, CD20-, PAX5+, CD79a+, CD5+ (focal), CD3-, CyclinD1+, CD45-, EBER-	CD45+, CD79a+, CyclinD1+, CD23-, CD30-, CD15-, PAX5+	Same rearrangement	Same rearrangement	NA	NA	On whole tissue sample: Clonal *IgH* rearrangement (whole tissue sample); abnormal caryotype: 47, XY,+3,del(6) (q15q21),t(11;14) (q13;q32)
Murray *et al.*, 2017 [72]	cHL-MC	Blastoid	Mixed	CD30+, CD15+, CD20+ (focal/weak), PAX5+, CD79a-, ALK1-, CyclinD1-, CD45-, EBER+, p53+, OCT2+, MUM1+	CD20+, CD45+, BCL2+BCL6-, CyclinD1+, CD10-, CD5+, CD23-, CD43+, p53-, PAX5+	Same rearrangement	Same rearrangement	NA	NA	On whole tissue sample: clonal rearrangement of *IgH* and *IgK*
Schneider *et al.,* 2014 [8]	cHL-LR	Classical	Mixed	CD30+, CD15+, CD20+ (focal), PAX5+, CD79a-, CD5-, CyclinD1+, SOX11+, CD45+ (focal), BCL2+, OCT2+	CD20+, CD45+, CD79a+BCL2-, CyclinD1+, SOX11+, CD5+, CD30-, CD15-, PAX5+, OCT2+	Same rearrangement	Same rearrangement	Common *IgH* rearrangement and *V* gene somatic hypermutations	Common *IgH* rearrangment and *V* gene somatic hypermutations	In both contingents: same *TP53* mutations (exon 5, Y163C) and *TP53/17p13* heterozygous deletions, no mutation of *SOCS1, TNFAIP3, NFKBIA, NOTCH1*

Sharma *et al.*, 2019 [73]	cHL	Classical	Mixed	CD30+, CD15+, CD20-, PAX5+, CD45-, BCL6-, LMP1+	CD20+, CD45+, BC2+, BCL6-, CyclinD1+, CD10-, CD5+, CD23-	NA	NA	NA	NA	NA
Tinguely *et al.*, 2003 [9]	cHL	Classical	Mixed	CD30+, CD15+, CD20+ (focal), CD3-, EBV+ (focal)LMP1+ (focal), BOB1+ (focal), OCT2+ (focal)	CD20+, CyclinD1+, EBER-, LMP1-, BOB1+, OCT2+	NA	NA	Common *IgH* rearrangement, presence of *V* gene somatic hypermutations	Common *IgH* rearrangement	NA

cHL: classical Hodgkin lymphoma; EBER: Epstein-Barr virus-encoded small RNA *in situ* hybridization; LR: lymphocyte rich; MC: Mixed-cellularity; MCL: Mantle-cell lymphoma; NA: Not available; NS: Nodular sclerosis; +: positive; -: negative.

Supplementary Table S6: clinical and laboratory data of cHL/marginal zone composite lymphomas.

References	Age (years)	Sex	History of lymphoma	Location	Tumor size (cm)	B symptoms	Ann Arbor stage	Other symptoms or tumor mass/LN	Laboratory data	Extension assessment	Treatment	Outcome	Follow-up duration (months)	Time of relapse / DOD (months)
Harada *et al.*, 2008 [75]	63	M	No	Retroperitoneal LN and spleen	Spleen: 980g	No	IV A	Fatigue	Anemia, LDH in normal range	CT-scan: Splenomegaly and retroperitoneal LN; BM+: SMZL	COP x3, then EPOCH etoposide, vincristine, adriamycin, cyclophosphamide and prednisone	Dead of sepsis	NA	NA
Zettl *et al.*, 2005 (case 1) [76]	54	M	NA	Stomach, Perigastric LN	NA	No	II	Weight loss, "gastric tumour"	NA	NA	Gastrectomy and CHOP x6	Complete response	106	NA
Zettl *et al.*, 2005 (case 2) [76]	53	M	No	Stomach, Perigastric LN	NA	No	II	Weight loss, "gastric tumour"	NA	NA	Gastrectomy and splenectomy and CHOP x6	Complete response	58	NA
Zettl *et al.*, 2005 (case 3) [76]	83	M	No	Stomach, Perigastric LN	NA	No	II	Weight loss, "gastric carcinoma"	NA	NA	Gastrectomy (no chemotherapy due to bad general conditions)	NA	9	NA
Zettl *et al.*, 2005 (case 4) [76]	76	M	No	Stomach, Perigastric LN	NA	No	II	Weight loss, "gastric tumour"	NA	NA	Gastrectomy and CHOP x4	Progressive disease and DOD at 7 months	7	7
Zettl *et al.*, 2005 (case 6) [76]	87	M	No	Stomach	NA	No	I	Weight loss, malaise	NA	NA	Gastrectomy and 5xCOPP/ABVD	Partial response and DOD at 48 months	48	NA
Zettl *et al.*, 2005 (case 7) [76]	82	F	No	Stomach, Inguinal LN	NA	No	III	NA	NA	NA	NA	NA	NA	NA
Zettl *et al.*, 2005 (case 8) [76]	84	M	No	Stomach Cervical LN	NA	No	III	Weight loss, malaise	NA	NA	HP radication	DOD (12 months)	12	12
Zettl *et al.*, 2005 (case 9) [76]	71	M	No	Bronchus	NA	No	III A	Cough, weight loss, lung tumor, mediastinal and para-aortal lymph nodes	NA	NA	CHOP x6 and rituximab	Complete response	4	NA
Zettl *et al.*, 2005 (case 10) [76]	58	F	No	Thyroid, cervical LN	NA	No	III A	Thyroid tumor	NA	NA	CHOP x6 and thyroidectomy	NA	2	NA
Zettl *et al.*, 2005 (case 11) [76]	67	F	Lymphoplasma cytoid immunocytoma in 1991, and DLBCL in 1998	Cervical LN	NA	No	NA	NA	NA	NA	Splenectomy, 6xCHOP and bendamustine	DOD (8 months)	8	8
Zettl *et al.*, 2005 (case 12) [76]	70	M	No	Inguinal LN	NA	Yes	III B	Cervical, axillary, mediastinal, abdominal, inguinal LN, splenomegaly	NA	NA	MCP HD9 x2 (German Hodgkin Study)	Partial remission and loss to follow-up	6	NA
Elmahy *et al.*, 2007 [77]	64	M	No	Cervical and axillary LN, spleen	NA	No	IV A	Abdominal pain and distension, night sweat	NA	CT-scan: extension to mesenteric, para-aortic LN, splenomegaly, moderate mediastinal LN and small axillary/pelvic LN; BM+: SMZL	ABVD x6 and then splenectomy	Complete remission	6	NA
Oka *et al.*, 2012 [74]	60	M	MALTL 2 years earlier	Stomach	18	No	NA	NA	NA	BM aspiration -	Gastrectomy and CHOP x3	Complete remission	131	122*
Oka *et al.*, 2010 [78]	66	F	No	Lung, stomach, and brain	2	No	IV A	Headaches and gait disturbance, cerebral tumor	NA	PET-scan: lung mass and systemic LN swelling	Gamma knife therapy x5, no chemotherapy, lung partial lobectomy	NA	86	43

Fung *et al.*, 2002 [79]	78	M	No	Mass of the left supraglottic larynx	3	No	I	Intermittent shortness of breath, progressive dysphagia, and intermittent hoarseness since 3 months due to large mass of the left supraglottic larynx that had a "ball-valve"	NA	NA	RT	Complete remission	31.2	NA
Aguilera *et al.*, 1996 [80]	74	M	No	Small bowel tumor and LN	9	No	NA	Acute abdomen perforation, periombilical pain due to small bowel tumor	NA	NA	No treatment	Dead from congestive heart failure	NA	NA
Maeshima *et al.,* 2015 [3]	45	M	No	Stomach, Paraaortic LN	NA	Na	II	NA	NA	NA	R-CHOP	Complete remission	79	NA
Demurtas *et al.*, 2011 (case 15) [60]	58	M	NA	Laterocervical LN	NA	Na	NA	NA	NA	NA	NA	NA	NA	NA
Schmitz *et al.*, 2005 (case 4) [6]	NA	NA	SMZL	NA	NA		NA	NA	NA	NA	NA	NA	NA	NA
Auditeau *et al.*, 2018 [81]	70	M	No	Hepato-splenomegaly, coeliac and mesenteric LN	NA	Yes	IV B	NA	Anemia, neutrophilia, lymphocytosis	CT-scan: hepato-splenomegaly, coeliac and mesenteric LN<br>BM+: MZL and cHL;<br>BM aspiration +: DLBCL and cHL	COP, ABVD and etoposide and then R-CHOEP x3	No remission and DOD	NA	NA

BM+/-: Involvement or not on bone marrow biopsy; BM aspiration +/-: Involvement or not on bone marrow aspiration; cHL: classical Hodgkin lymphoma; CT-scan: computed tomography-scan; DLBCL: Diffuse large B-cell lymphoma; DOD: Dead of disease; F: Female; HP: *Helicobacter pylori*; LDH: Lactate dehydrogenase; LN: Lymph node(s); M: Male; MALTL: Mucosa-associated lymphoid tissue lymphoma; MZL: Marginal zone lymphoma; NA: Not available; PET-scan: Positron emission tomography-scan; RT: Radiotherapy; SMZL: Splenic marginal zone lymphoma. For chemotherapy abbreviations, please see **Supplementary Text S1**.

*Not proved by histopathology (CT-scan only).

Supplementary Table S7: histopathological and molecular data of cHL/marginal zone composite lymphomas.

References	cHL type	MZL type	Separated / mixed contingents	Immunophenotype cHL	Immunophenotype MZL	t(11;18) (cHL)	t(11;18) (MZL)	Clonal B rearrangements (cHL)	Clonal B rearrangements (MZL)	Other molecular alterations
Harada *et al.*, 2008 [75]	cHL	SMZL	Mixed	CD30+, CD15+ (weak), CD20-, PAX5+, CD45-, BOB1-, OCT2-	CD20+, BCL2+, CD5-, CD30-, CD15-	NA	NA	NA	NA	On whole tissue sample: clonal rearrangement of *IgH*
Zettl *et al.*, 2005 (case 1) [76]	cHL-MC	MALTL + DLBCL	Separated (synchronous)	CD30+, CD15-, CD20-, CD79a-, K/L light chains-, CD45-, BCL2+ (focal), EBER-, LMP1-, p53+, MUM1+	CD20+, CD45+, CD79a+, K/L light chains-, BCL2+, p53-, CD30-, CD15-, EBER-, LMP1-, MUM1+ (focal)	NA	NA	NA	NA	NA
Zettl *et al.*, 2005 (case 2) [76]	cHL-MC	MALTL + DLBCL	Separated (synchronous)	CD30+, CD15+ (focal), CD20+ (focal), CD79a-, K/L light chains-, CD45-, BCL2-, EBER+, LMP1+, p53+, MUM1+	CD20+, CD45+, CD79a+, K/L light chains-, BCL2+, p53-, CD30-, CD15-, EBER-, LMP1-, MUM1-	NA	NA	NA	NA	NA
Zettl *et al.*, 2005 (case 3) [76]	cHL-MC	MALTL + DLBCL	Separated (synchronous)	CD30+, CD15+, CD20+ (focal), CD79a-, -, K/L light chains-, CD45-, BCL2+, EBER-, LMP1-, p53+, MUM1+	CD20+, CD45+, CD79a+, K/L light chains-, BCL2+, p53-, CD30-, CD15-, EBER-, LMP1-, MUM1-	NA	NA	NA	NA	NA
Zettl *et al.*, 2005 (case 4) [76]	cHL-MC	MALTL + DLBCL	Separated (synchronous)	CD30+, CD15-, CD20+, CD79a-, K/L light chains-, CD45-, BCL2+, EBER+, LMP1+, p53+, MUM1+	CD20+, CD45+, CD79a+, K light chain+, L light chain-, BCL2+, p53-, CD30-, CD15-, EBER-, LMP1-, MUM1+ (focal)	NA	NA	NA	NA	NA
Zettl *et al.*, 2005 (case 6) [76]	cHL	MALTL	NA	CD30+, CD15+, CD20-, K/L light chains-, CD45-, LMP1-	CD20+, CD45+, +, K light chain+, L light chain-, CD30-, CD15-, EBER-, LMP1-	NA	-	NA	NA	NA
Zettl *et al.*, 2005 (case 7) [76]	cHL-MC	MALTL	NA	CD30+, CD15+CCD20+, CD79a-, K/L light chains-, CD45-, BCL2+, EBER-, LMP1-, p53+, MUM1+	CD20+, CD45+, CD79a, +, K light chain+, L light chain-, BCL2+, p53-, CD30-, CD15-, EBER-, LMP1-, MUM1+ (focal)	NA	-	NA	NA	NA
Zettl *et al.*, 2005 (case 8) [76]	cHL-MC	MALTL	NA	CD30+, CD15+, CD20-, K/L light chains-, CD45-, EBER-, LMP1-	CD20+, CD45+, K/L light chains-, CD30-, CD25-, EBER-, LMP1-	NA	-	NA	NA	NA
Zettl *et al.*, 2005 (case 9) [76]	cHL	MALTL	NA	CD30+, CD15-, CD20+ (focal), CD79a-, K/L light chains-, CD45-, BCL2+, LMP1-	CD20+, CD45+, CD79a+, K/L light chains-, BCL2-, p53-, CD30-, CD15-, EBER-, LMP1-	NA	-	NA	NA	NA
Zettl *et al.*, 2005 (case 10) [76]	cHL-MC	MALTL + DLBCL	Separated (synchronous)	CD30+, CD15+, CD20-, CD79a-, K/L light chains-, CD45-, BCL2+, EBER+, LMP1+, p53+, MUM1+	CD20+, CD45+, CD79a+, K/L light chains-, BCL2+, p53-, CD30-, CD15-, EBER-, LMP1-, MUM1-	NA	NA	NA	NA	NA
Zettl *et al.*, 2005 (case 11) [76]	cHL-MC	MZL	NA	CD30+, CD15+ (focal), CD20+, CD79a-, K/L light chains-, CD45-, BCL2+, EBER+, LMP1+, p53+, MUM1+	CD20+, CD45+, CD79a, +, K light chain+, L light chain-, BCL2+, p53-, CD30-, CD15-, EBER-, LMP1-, MUM1+ (focal)	NA	-	NA	NA	NA
Zettl *et al.*, 2005 (case 12) [76]	cHL-MC	MZL	NA	CD30+, CD15-, CD20+ (focal), CD79a-, K/L light chains-, CD45-, BCL2+, EBER+, LMP1+	CD20-, CD45-, CD79a+, K light chain-, L light chain+, BCL2+, CD30-, CD15-, EBER-, LMP1-	NA	-	NA	NA	NA
Elmahy *et al.*, 2007 [77]	cHL-MC	SMZL	NA	CD30+, CD15+	CD20+, K light chain-, L light chain+, CyclinD1-, IgD+, IgM+, CD5-	NA	NA	NA	NA	NA
Oka *et al.,* 2012 [74]	cHL	MALTL	Mixed	CD30+, CD15+, CD20+ (focal/weak), PAX5-, CD79a+ (focal/weak), ALK1-, CD5-, CD3-, CyclineD1-, CD45-, EBER+, LMP1+, EMA-, BOB1-, OCT2-	CD20-, CD45-, CD79a-, CyclineD1-, CD5-, CD3-, CD30-, CD15-, EBER-, LMP1-, PAX5+, BOB1+, OCT2-	NA	NA	NA	NA	Bone marrow: Chromosome analysis: 47, XY, +der(17) t(1; 17)(q21; q25)

Oka *et al.,* 2010 [78]	cHL-NS	MALTL	Separated (synchronous)	CD30+, CD15+, CD20+, CD79a-, CD45-, EBER+, LMP1+, BOB1-, OCT2-	CD20+, CD79a+, CD3-, CD30-, CD15-, EBER+	NA	NA	NA	NA	NA
Fung *et al.,* 2002 [79]	cHL	MALTL	Mixed	CD30+, CD15+, CD20+ (focal), CD45-, LMP1-	CD20+, CD45+, CD79a+, BCL2+ (focal)	NA	NA	NA	NA	NA
Aguilera *et al.,* 1996 [80]	cHL-MC	MZL	Separated	CD30+, CD15+, CD20-, CD45-, LMP1+	CD20+, CD45+, K/L light chains not helpfull, LMP1+	NA	NA	NA	NA	NA
Maeshima *et al.,* 2015 [3]	cHL-MC	MALTL + DLBCL	Separated (synchronous)	CD30+, CD15-, CD20-, PAX5+, CD79a-, EBER-, BOB1-, OCT2-	CD20+, CD10-, CD5-CD30+ (focal), EBER-	-	-	NA	NA	No *BCL2* translocation in both contingents
Demurtas *et al.,* 2011 (case 15) [60]	cHL	MZL	NA	NA	CD10-, CD5-, CD23- (cytometry)	NA	NA	NA	NA	On whole tissue sample: clonal rearrangement of *IgH*
Schmitz *et al.,* 2005 (case 4) [6]	cHL	SMZL	Separated (synchronous)	NA	NA	NA	NA	Absence of *IgH* rearrangement or *V* gene mutation	Absence of *IgH* rearrangement or *V* gene mutation	Absence of *FAS*, *NFKBIA* and *ATM* mutation
Auditeau *et al.,* 2018 [81]	cHL	DLBCL and MZL	NA	CD30+, CD15+, CD20-, CD79a-, ALK1-, CD5-, CD3-,	CD20+, CD10-, CD5- (cytometry)	NA	NA	NA	NA	NA

cHL: classical Hodgkin lymphoma; DLBCL: Diffuse large B-cell lymphoma; EBER: Epstein-Barr virus-encoded small RNA *in situ* hybridization; MALTL: Mucosa-associated lymphoid tissue lymphoma; MC: Mixed cellularity; MZL: Marginal zone lymphoma; NA: Not available; NS: Nodular sclerosis; SMZL: Splenic marginal zone lymphoma; +: positive; -: negative.

Supplementary Table S8: clinical and laboratory data of cHL/DLBCL composite lymphomas.

References	Age (years)	Sex	History of lymphoma	Location	Tumor size (cm)	B symptoms	Ann Arbor stage	Other symptoms or tumor mass/LN	Laboratory data	Extension assessment	Treatment	Outcome	Follow-up duration (months)	Time of relapse / DOD (months)
Gonzales *et al*., 1991 (case 1) [14], Kingma *et al*., 1994 (case 1) [66]	66	M	No	Cervical LN	NA	No	IV A	NA	NA	NA	Cyclophosphamide, vincristine, bleomycin	NA	NA	NA
Gonzales *et al*., 1991 (case 2) [14], Kingma *et al*., 1994 (case 2) [66]	43	M	DLBCL 6 years earlier, FL 4 years earlier	Inguinal and supraclavicular LN	NA	No	NA	NA	NA	NA	Multiagent chemotherapy and ASCT	DOD 26 months after the diagnosis	26	26
Gonzales *et al*., 1991 (case 3) [14], Kingma *et al*., 1994 (case 3) [66]	63	M	FL 3 years earlier	Stomach	NA	No	NA	NA	NA	NA	Surgery only	DOD 2 months after diagnosis	2	2
Goonzales *et al*., 1991 (case 4) [14]	62	F	No	Inguinal LN	NA	No	I A	NA	NA	NA	Local RT	Complete remission	27	NA
Gonzales *et al*., 1991 (case 5) [14], Kingma *et al*., 1994 (case 4) [66]	22	M	No	Cervical LN	NA	Yes	IV B	NA	NA	NA	Cyclophsophamide, cisplatinium, prednisone, carmustine	DOD 6 months after diagnosis	6	6
Gonzales *et al*., 1991 (case 7) [14], Kingma *et al*., 1994 (case 6) [66]	25	M	No	Mediastinum	NA	No	II A	NA	NA	NA	Multiagent chemotherapy	Complete remission	3	NA
Kingma *et al.,* 1994 (case 5) [66]	84	F	No	Cervical LN	NA	NA	NA	NA	NA	NA	NA	NA	NA	NA
Kingma *et al*., 1994 (case 9) [66]	70	F	No	Inguinal LN	NA	NA	NA	NA	NA	NA	NA	NA	NA	NA
Kingma *et al*., 1994 (case 10) [66]	63	M	No	LN (unspecified)	NA	NA	NA	NA	NA	NA	NA	NA	NA	NA
Kingma *et al*., 1994 (case 11) [66]	58	M	No	Cervical LN	NA	NA	NA	NA	NA	NA	NA	NA	NA	NA
Kingma *et al*., 1994 (case 12) [66]	66	F	No	Axillary LN	NA	NA	NA	NA	NA	NA	NA	NA	NA	NA
Kingma *et al*., 1994 (case 1*) [66]	76	F	No	LN (unspecified) and small intestine	NA	NA	NA	NA	NA	NA	NA	NA	NA	NA
Kingma *et al.,* 1994 (case 2*) [66]	16	F	No	Cervical LN and ovary	NA	NA	NA	NA	NA	NA	NA	NA	NA	NA
Nishioka *et al.,* 2018 [64]	73	F	No	Systemic LN and ulcerative mass of the esophagus	1,5	Yes	IV B	NA	Soluble IL-2R and LDH increase	PET-scan: cervical, axillary, mediastinal, supraclavicular, para-aortic, and inguinal LN	8x R-CHOP, and DeVIC x4 for the relapse	Complete remission, then, relapse (1 month later on cHL), then complete remission	1	1

										Esophagogastroduodenoscopy: ulcerative mass in the mid-esophagus;<br>BM+ (*IgH* rearrangment identified, whitout tumor cell vizualised)				
Zettl *et al.*, 2005 (case 1) [76]	54	M	No	Stomach and perigastric LN	NA	No	II E	Weight loss, gastric tumor	NA	NA	Gastrectomy and CHOP x6	Complete response	106	NA
Zettl *et al.*, 2005 (case 2) [76]	53	M	No	Stomach and perigastric LN	NA	No	II E	Weight loss, gastric tumor	NA	NA	Gastrectomy and splenectomy and CHOP x6	Complete response	58	NA
Zettl *et al.*, 2005 (case 3) [76]	83	M	No	Stomach and perigastric LN	NA	No	II E	Weight loss, gastric tumor	NA	NA	Gastrectomy (no chemotherapy due to bad general conditions)	NA	9	NA
Zettl *et al.*, 2005 (case 4) [76]	76	M	No	Stomach and perigastric LN	NA	No	II E	Weight loss, gastric tumor	NA	NA	Gastrectomy and CHOP x4	Progressive disease and DOD at 7 months	7	7
Zettl *et al.*, 2005 (case 5) [76]	73	F	No	Stomach and perigastric LN	NA	No	II E	NA	NA	NA	NA	NA	NA	NA
Zettl *et al.*, 2005 (case 10) [76]	58	F	No	Thyroid and cervical LN	NA	No	II A E	Thyroid tumor	NA	NA	CHOP x6 and thyroidectomy	NA	2	NA
Geladari *et al.*, 2020 [82]	73	F	NA	Cervical, axillary, hepato-gastric, pancreaticoduodenal and paraaortic LN, splenomegaly,	NA	Yes	III B	Abdominal fullness and icterus since 2 days with pain after meals. Pruritus since 2 months	Liver and cholestatic enzyme increase	NA	NA	NA	NA	NA
Schmitz *et al.*, 2005 (case 6) [6]	NA	NA	NA	NA	NA	NA	NA	NA	NA	NA	NA	NA	NA	NA
Maeshima *et al.,* 2015 [3]	45	M	No	Stomach Paraaortic LN	NA	NA	II	NA	NA	NA	R-CHOP	Complete remission	79	NA
Aussedat *et al.*, 2020 (Com-22) [16]	NA	NA	NA	Supraclavicular LN	NA	NA	NA	NA	NA	NA	R-ACVBP, MTX, R-DHAOx, IVOX, ASCT, RT	NA	NA	NA
Aussedat *et al.*, 2020 (Com-101) [16]	NA	NA	NA	Cervical LN	NA	NA	NA	NA	NA	NA	DLBCLR-CHOP	NA	NA	NA
Aussedat *et al.*, 2020 (Com-158) [16]	NA	NA	NA	Tonsil and mesenteric LN	NA	NA	NA	NA	NA	NA	R-CHOP, R-ABVD	NA	NA	NA
Aussedat *et al.*, 2020 (Com-219) [16]	NA	NA	NA	Mediastinum	NA	NA	NA	NA	NA	NA	DLBCL R-COPADM, CYM	NA	NA	NA
Aussedat *et al.*, 2020 (Com-236) [16]	NA	NA	NA	Subcutaneous	NA	NA	NA	NA	NA	NA	escBEACOPP	NA	NA	NA
Aussedat *et al.*, 2020 (Com-240) [16]	NA	NA	NA	Supraclavicular LN, stomach and mediastinum	NA	NA	NA	NA	NA	NA	R-ACVBP	NA	NA	NA

Aussedat *et al.*, 2020 (Com-251) [16]	NA	NA	NA	Inguinal LN	NA	NA	NA	NA	NA	NA	R-CHOP	NA	NA	NA
Aussedat *et al.*, 2020 (Com-253) [16]	NA	NA	NA	Mediastinum	NA	NA	NA	NA	NA	NA	R-COPADM, CYM	NA	NA	NA
Aussedat *et al.*, 2020 (Com-312) [16]	NA	NA	NA	Mediastinum	NA	NA	NA	NA	NA	NA	R-ACVBP, BV-ICE, ASCT	NA	NA	NA
Wilson *et al.*, 2014 [83]	NA	NA	NA	NA	NA	NA	NA	NA	NA	NA	NA	NA	NA	NA
Bellan *et al.*, 2002 [84]	29	F	No	Cervical, supraclavicular LN	NA	No	NA	No	NA	NA	MACOP-B and ASCT, RT	Complete remission	30	NA
Rosenquist *et al.*, 2004 [13]	74	M	No	Retroperitonal mass and abdominal, inguinal LN	12	Yes	IV B	No	NA	BM+: cHL	R-CHOP	Complete remission	12	NA
Wei *et al.,* 2004 [85]	26	F	No	Mediastinal	NA	NA	NA	Chest pain and night sweats	NA	BM-	R-CHOP et RT	NA	NA	NA
Traverse-Glehen *et al.*, 2005 (case 22) [86]	46	M	NA	Mediastinum	NA	NA	NA	NA	NA	NA	NA	NA	NA	NA
Traverse-Glehen *et al.*, 2005 (case 23) [86]	25	M	NA	Mediastinum and liver	NA	NA	NA	NA	NA	NA	NA	NA	NA	NA
Traverse-Glehen *et al.*, 2005 (case 24) [85]	25	M	NA	Mediastinum	NA	NA	NA	NA	NA	NA	NA	NA	NA	NA
Traverse-Glehen *et al.*, 2005 (case 25) [86]	31	F	NA	Mediastinum	NA	NA	NA	NA	NA	NA	NA	NA	NA	NA
Traverse-Glehen *et al.*, 2005 (case 26) [86]	21	M	NA	Mediastinum	NA	NA	NA	NA	NA	NA	NA	NA	NA	NA
Traverse-Glehen *et al.*, 2005 (case 27) [86]	40	M	NA	Mediastinum and cervical LN	NA	NA	NA	NA	NA	NA	NA	NA	NA	NA
Quintanilla-Martinez *et al.*, 2009 [87]	NA	F	NA	Mediastinum	NA	NA	NA	NA	NA	NA	NA	NA	NA	NA
Quintanilla-Martinez *et al.*, 2009 [87]	NA	F	NA	Mediastinum	NA	NA	NA	NA	NA	NA	NA	NA	NA	NA
Quintanilla-Martinez *et al.*, 2009 [87]	NA	F	NA	Mediastinum	NA	NA	NA	NA	NA	NA	NA	NA	NA	NA
Quintanilla-Martinez *et al.*, 2009 [87]	NA	F	NA	Mediastinum	NA	NA	NA	NA	NA	NA	NA	NA	NA	NA

Oschlies *et al.*, 2011 [88]	NA	NA	NA	NA	NA	NA	NA	NA	NA	NA	NA	NA	NA	NA
Eberle *et al.*, 2011 [89]	23	M	NA	NA	NA	NA	NA	NA	NA	NA	NA	NA	NA	NA
Yu *et al.*, 2011 [90]	37	F	No	Bilateral supraclavicular LN	7	No	NA	Shoulder and chest pain since 3 months	Anemia, tumor specific growth factor increase	Abdominal ultrasonography: no abdormalities	R-CHOP x6 and RT	Complete remission	33	NA
Wang *et al.*, 2013 [91]	53	F	No	Gastric ulcers (pylorus and corpus), and perigastric, parapyloric LN	2.5 to 4	No	VI A	Upper abdominal discomfort and flatulent pain since 8 months, weight loss and anorexia since 6 months	Anemia	Gastroscopy: 2 ulcers of the gastric corpus and mucosal nodularities;<br>CT-scan : no other LN	CHOP x8 and R-CHOP x2 after relapse	Relapse (unspecified) 7 months after chemotherapy, DOD at 11 months	11	7
Khan *et al.*, 2017 [92]	18	F	No	Right pulmonary mass with liver lesions and multiple small masses in both kidneys	14	No	VI A	Syncopes, swelling of neck and face since 3 weeks (superior vena cava syndrome)	Anemia, LDH increase	BM-	EPOCH-R x8 and intrathecal methotrexate	Complete remission	NA	NA
Goyal *et al.*, 2016 [93]	79	F	NA	Ileum mass and right colon mass and mesenteric LN	11 and 0,8	NA	NA	NA	NA	NA	R-CHOP x6	Complete remission	6	NA
Khanna *et al.*, 2010 [94]	58	M	DLBCL 3 years earlier	Spleen, splenic hilar/peripancreatic LN, mitral and aortic valve masses	1.1 and 2.2	No	VI A	NA	NA	PET-scan: lesion of the spleen and splenic hilar LN; Echocardiography: mass of the mitral and aortic valves	Cardiac surgery and R-CHOEP x6, bleomycin, methotrexate, cytarabine	Complete remission	7	NA
Auditeau *et al.*, 2018 [81]	70	M	No	Hepato-splenomegaly, coeliac and mesenteric LN	NA	Yes	IV B	NA	Anemia, neutrophilia, lymphocytosis	Ct scan : hepato-splenomegaly, coeliac and mesenteric LN<br>BM+: MZL and cHL;<br>BM aspiration +: DLBCL and cHL	COP, ABVD, etoposide and then R-CHOEP x3	No remission and DOD	NA	NA
Casey *et al.*, 1990 (case 1) [95]	27	M	No	Mediastinum	NA	NA	NA	NA	NA	NA	NA	NA	NA	NA
Casey *et al.*, 1990 (case 8) [95]	64	M	No	Mesenteric LN and liver/spleen	NA	NA	IV	NA	NA	NA	NA	NA	NA	NA
Guarner *et al.*, 1990 [96]	44	M	No (HIV+)	Cervical, axillary, periaortic, retroperitoneal LN and liver/spleen	1	No	IV A	Fever (40°) and chills, severe diarrhea		BM: multiple non-caseating granulomas	ProMACE/MOPP	Dead of gastrointestinal hemorrhage and bacteremia after 6 months, invasive aspergilossis, and CMV infection	NA	NA
Huang *et al.*, 2006 [97]	56	M	cHL-NS, 12 years earlier, relapse 9 years earlier	Generalized LN	4	Yes	IV B	No	NA	CT-scan: multiple pulmonary nodules, hepatic lesions, mediastinal, porta hepatis, retroperitoneal and mesenteric LN, small intestine tumor;<br>BM+ (cHL)	Dead of sepsis, acute renal failure, liver failure, coagulopathy 20 days after the admission	NA	0.67	NA

Wang *et al.*, 2020 [98]	64	M	No	Cervical LN (bilateral)	2.5	No	II A	No	NA	NA	R-CHOP x6 and R in maintenance therapy	Complete remission	6	NA
Kerl *et al.,* 1999 [99]	66	NA	NA	Lung	NA	NA	NA	NA	NA	NA	NA	NA	NA	NA
Perwein *et al.*, 2020 (case 4) [100]	14	F	No	Mediastinal, cervical and infraclavicular LN, pleural and pericardial effusion, chest wall, pulmonary, hepatic, pancreatic involvements		No	III A E	Thoracic pain, tacchycardia	LDH in normal range	NA	Dose adjusted-EPOCH-R x6 and for relapse IEP x2 + COPDAC x2 + brentuximab x2, HD-BEAM + ASCT	Complete remission for 3.9 years and then relapse (cHL) and then complete remission after second line chemotherapy	88.8	46.8
Perwein *et al.*, 2020 (case 1) [100]	17	F	No	Mediastinal, cervical, supraclavicular, abdominal LN	NA	No	III A	Neck and face swollen, superior vena cava syndrome	LDH increase, hyperleucocytosis	NA	BFM 04 protocol and RT for first line, and R + Hodgkin-like therapy but partial remission; For second line: DXM+BEAM and R-COP x2, HD-BEAM + ASCT + RT	Complete remission but relapse (cHL), and complete remission after second line therapy	124.8	5
Miyagaki *et al.*, 2019 [101]	75	M	cHL 13 years earlier and mycosis fungoid 11 years earlier	Subcutaneous abdominal mass and aortic bifurcation / inguinal LN	3.5	Yes	II B	No	NA	NA	R-CHOP	NA	36	NA
Paulli *et al.,* 1992 [102]	37	M	No	Supraclavicular, mediastinal and abdominal LN	NA	No	IV A	Fever and malaise since 4 weeks	Anemia, elevation of α-2 globulin, low serum albumin, LDH in normal range	BM+: cHL	pro-MACE-CytaBOM x8	Complete remission	23	NA
Hwang *et al.*, 2011 [103]	58	M	NA	Multiple LN and splenomegaly	15 (spleen)	Yes	IV B	No	Anemia, circulating EBV DNA	BM+: DLBCL	R-ABVD, intrathecal methotrexate after central nervous system relapse	Complete remission after 3 courses of treatment but isolated central nervous system lymphoma relapse (DLBCL, EBV DNA in cerebrospinal fluid) and died of sepsis	NA	NA
Hell *et al.*, 1995 (case 3) [104]	NA	NA	NA	LN	NA	NA	NA	NA	NA	NA	NA	NA	NA	NA
Hell *et al.*, 1995 (case 6) [104]	NA	NA	NA	LN	NA	NA	NA	NA	NA	NA	NA	NA	NA	NA
Soliman *et al.*, 2016 [105]	60	F	No	Cervical LN and hepato-splenomegaly	NA	Yes	IV B	Fatigue, bilateral lower limb pain	LDH increase, low albuminal rate, hepatic cytolysis, elevated alkaline phosphatase, seric EBV DNA, Anemia	BM aspiration on biopsy+: Burkitt lymphoma/leukemia (27%); MRI: involvement of skull base and axial skeleton; PET-scan: cervical, supraclavicular, mediastinal, retroperitoneal, multiple liver/spleen and skeletal muscle lymphomatous	R-CONDOX-M/R-IVAC (intrathecal) + RT + R x2	Complete remission	NA	NA

										lesions, multiple pleural, peritoneal, extensive gastric and sporadic bowel involvement, multiple bone, bilateral breast and right adrenal foci involvement				
Kim *et al.,* 1977 (case 18) [1]	40	M	NA	Spleen, splenic hilar, axillary, hepatoduodenal LN (CHL-NS)	Spleen : 866g	NA	NA	NA	NA	NA	Chemotherapy and RT	Complete remission but DOD 54 months after the diagnosis	54	54
Jaffe *et al.,* 1992 (case 3) [58]	43	M	No	Inguinal and spuraclavicular LN	NA	NA	NA	NA	NA	NA	NA	NA	NA	NA
Jaffe *et al.,* 1992 (case 6) [58]	25	M	No	Mediastinum	NA	NA	NA	NA	NA	NA	NA	NA	NA	NA
Jaffe *et al.,* 1992 (case 7) [58]	63	M	No	Stomach	NA	NA	NA	NA	NA	NA	NA	NA	NA	NA
Jaffe *et al.,* 1992 (case 8) [58]	66	M	No	Cervical LN	NA	NA	NA	NA	NA	NA	NA	NA	NA	NA
Jaffe *et al.,* 1992 (case 9) [58]	22	M	No	Cervical LN	NA	NA	NA	NA	NA	NA	NA	NA	NA	NA
Jaffe *et al.,* 1992 (case 10) [58]	73	M	No	Inguinal LN	NA	NA	NA	NA	NA	NA	NA	NA	NA	NA
Jaffe *et al.,* 1992 (case 11) [58]	70	M	No	Inguinal LN	NA	NA	NA	NA	NA	NA	NA	NA	NA	NA
Jaffe *et al.,* 1992 (case 12) [58]	63	M	No	LN (unspecified)	NA	NA	NA	NA	NA	NA	NA	NA	NA	NA
Jaffe *et al.,* 1992 (case 14) [58]	76	F	No	Small bowel and mesenteric LN	NA	NA	NA	NA	NA	NA	NA	NA	NA	NA
Jaffe *et al.,* 1992 (case 15) [58]	16	F	No	Ovary LN and spleen	NA	NA	NA	NA	NA	NA	NA	NA	NA	NA

BM+/-: Involvement or not on bone marrow biopsy; BM aspiration +/-: Involvement or not on bone marrow aspiration; cHL: classical Hodgkin lymphoma; CT-scan: computed tomography-scan; DLBCL: Diffuse large B-cell lymphoma; DNA: Desoxyribonucleic acid; DOD: Dead of disease; EBV: Epstein-Barr Virus; F: Female; FL: Follicular lymphoma; HIV: Human immunodeficiency virus; IL-2R: Interleukine 2 receptor; LDH: Lactate dehydrogenase; LN: Lymph node(s); M: Male; NA: Not available; NS: Nodular sclerosis; PET-scan: Positron emission tomography-scan; RT: Radiotherapy. For chemotherapy abbreviations, please see **Supplementary Text S1**.

*Case from the study of Kingma et al., 1994 [65] in the synchronous/simultaneous lymphomas table.

Supplementary Table S9: histopathological and molecular data of cHL/DLBCL composite lymphomas.

References	cHL type	DLBCL type	Separated / mixed contingents**	Immunophenotype cHL	Immunophenotype DLBCL	Clonal B rearrangements (cHL)	Clonal B rearrangements (DLBCL)	Other molecular alterations
Gonzales *et al*., 1991 (case 1) [14], Kingma *et al*., 1994 (case 1) [66]	cHL-NS	DLBCL	NA (DLBCL, 75%)	CD15+, CD20-, CD45-, UCHL1-, EBER-	CD20+, CD45+, UCHL1-, CD15-, EBER-	NA	NA	NA
Gonzales *et al*., 1991 (case 2) [14], Kingma *et al*., 1994 (case 2) [66]	cHL-NS	DLBCL	NA (DLBCL, 90%)	CD15+, CD20-, CD45-, UCHL1-, EBER-	CD20+, CD45+, UCHL1-, CD15-, EBER-	NA	NA	NA
Gonzales *et al*., 1991 (case 3) [14], Kingma *et al*., 1994 (case 3) [66]	cHL-MC	DLBCL	NA (DLBCL, 90%)	CD15+, CD20-, CD45-, UCHL1-, EBER+	CD20+, CD45+, UCHL1-, CD15-, EBER+	NA	NA	NA
Gonzales *et al*., 1991 (case 4) [14]	cHL-NS	DLBCL	NA, equal quantity	NA	NA	NA	NA	NA
Gonzales *et al*., 1991 (case 5) [14], Kingma *et al*., 1994 (case 4) [66]	cHL-MC	DLBCL	NA (DLBCL, 75%)	EBER-	CD20+, CD45+, UCHL1-, CD15-, EBER-	NA	NA	NA
Gonzales *et al*., 1991 (case 7) [14], Kingma *et al*., 1994 (case 6) [66]	cHL-NS	DLBCL	NA, equal quantity	EBER+	CD20+, CD45+, UCHL1-, CD15-, EBER+	NA	NA	NA
Kingma *et al.,* 1994 (case 5) [66]	cHL	DLBCL	NA	CD15+, CD20-, CD45-, UCHL1-, EBER+	CD20+, CD45+, UCHL1-, CD15-, EBER+	NA	NA	NA
Kingma *et al*., 1994 (case 9) [66]	cHL-NS	DLBCL	NA	CD15-, CD20+, CD45-, UCHL1-, EBER+	CD20+, CD45+, UCHL1-, CD15-, EBER+	NA	NA	NA
Kingma *et al*., 1994 (case 10) [66]	cHL	DLBCL	NA	CD15+, CD20+, CD45-, UCHL1-, EBER-	CD20+, CD45+, UCHL1-, CD15-, EBER-	NA	NA	NA
Kingma *et al*., 1994 (case 11) [66]	cHL	DLBCL	NA	CD15+, CD20-, CD45-, UCHL1-, EBER-	CD20+, CD45+, UCHL1-, CD15-, EBER-	NA	NA	NA
Kingma *et al*., 1994 (case 12) [66]	cHL-MC	DLBCL	NA	CD15-, CD20-, CD45-, UCHL1-, EBER+	CD20+, CD45+, UCHL1-, CD15-, EBER-	NA	NA	NA
Kingma *et al*., 1994 (case 1*) [66]	cHL-MC	DLBCL	Separated (synchronous)	CD15+, CD20-, CD45-, UCHLA-, EBER-	CD20+, CD45+, UCHL1-, CD15-, EBER-	NA	NA	NA
Kingma *et al.,* 1994 (case 2*) [66]	cHL	DLBCL	Separated (synchronous)	CD15-, CD20-, CD45-, UCHLA-, EBER-	CD20+, CD45+, UCHL1-, CD15-, EBER-	NA	NA	NA
Nishioka *et al.,* 2018 [64]	cHL	DLBCL	Separated (synchronous)	CD30+, CD15+, EBER-	CD20+, BCL2+, BCL6-, CD10-, CD3-, MUM1+	Clonal *IgH* rearrangement	Rearrangement not confirmed (not enough DLBCL sample)	On whole tissue sample: complex karyotype t(14:18)(q32:p21), and t(14:18) (*BCL2/IgH* translocation)
Zettl *et al.,* 2005 (case 1) [76]	cHL-MC	DLBCL	Separated (synchronous)	CD30+, CD15-, CD20-, CD79a-, K/L light chains-, CD45-, BCL2+ (focal), EBER-, LMP1-, p53+, MUM1+	CD20+, CD45+, CD79a+, BCL2+, BCL6-, p53-, CD10-, CD30-, CD15-, LMP1-, MUM1+	NA	NA	NA
Zettl *et al.,* 2005 (case 2) [76]	cHL-MC	DLBCL	Separated (synchronous)	CD30+, CD15+ (focal), CD20+ (focal), CD79a-, K/L light chains-, CD45-, BCL2-, EBER+,	CD20+, CD45+, CD79a+, BCL2+, BCL6-, p53-, CD10-, CD30-, CD15-, EBER-, LMP1-, MUM1+	NA	NA	NA

				LMP1+, p53+, MUM1+				
Zettl *et al.,* 2005 (case 3) [76]	cHL-MC	DLBCL	Separated (synchronous)	CD30+, CD15+, CD20+ (focal), CD79a-, -, K/L light chains-, CD45-, BCL2+, EBER-, LMP1-, p53+, MUM1+	CD20+, CD45+, CD79a+, BCL2+, BCL6-, p53-, CD10-, CD30+ (focal),CD15-, EBER-, LMP1-, MUM1+	NA	NA	NA
Zettl *et al.,* 2005 (case 4) [76]	cHL-MC	DLBCL	Separated (synchronous)	CD30+, CD15-, CD20+, CD79a-, K/L light chains-, CD45-, BCL2+, EBER+, LMP1+, p53+, MUM1+	CD20-, CD45+, CD79a+, BCL2+, BCL6-, p53+, CD10-, CD30-, CD15-, EBER-, LMP1-, MUM1+	NA	NA	NA
Zettl *et al.,* 2005 (case 5) [76]	cHL-MC	DLBCL	Separated (synchronous)	CD30+, CD15-, CD20-, CD45-, CD79a-, BCL2+, EBER-, LMP1-, p53+, MUM1+	CD20+, CD45+, CD79a+, BCL2+, BCL6-, p53-, CD10-, CD30-, CD15-, EBER-, LMP1-, MUM1-	NA	NA	NA
Zettl *et al.,* 2005 (case 10) [76]	cHL-MC	DLBCL	Separated (synchronous)	CD30+, CD15+, CD20-, CD79a-, K/L light chains-, CD45-, BCL2+, EBER+, LMP1+, p53+, MUM1+	CD20+, CD45+, CD79a+, BCL2+, BCL6-, p53-, CD10-, CD30-, CD15-, EBER-, LMP1-, MUM1-	NA	NA	NA
Geladari *et al.*, 2020 [82]	cHL	DLBCL	NA	CD30+, CD15+, CD20-, PAX5+, EBER+	CD20+, PAX5+, Ki67 80%	NA	NA	NA
Schmitz *et al*., 2005 (case 6) [6]	cHL	DLBCL	NA	EBER-	EBER-	Same *IgH* rearrangement, shared and distinct *V* gene somatic hypermutations	Same *IgH* rearrangement, shared and distinct *V* gene somatic hypermutations	*TP53* variant identifier on the DLBCL contingent only
Maeshima *et al.,* 2015 [3]	cHL-MC	DLBCL	Separated (synchronous)	CD30+, CD15-, CD20-, PAX5+, CD79a-, EBER-, BOB1-, OCT2-	CD20+, CD10+, CD30+ (focal), EBER-, CD5-	NA	NA	No *BCL2* or *MALT1* translocation in both contingents
Aussedat *et al*., 2020 (Com-22) [16]	cHL-NS	PMBL	NA	CD30+, EBER-	CD20+, EBER-	NA	NA	NA
Aussedat *et al*., 2020 (Com-101) [16]	cHL-NS	DLBCL	NA	CD30+, EBER-	CD20+, EBER-, CD30-	NA	NA	NA
Aussedat *et al*., 2020 (Com-158) [16]	cHL-NS	DLBCL	Separated (synchronous)	CD30+, EBER-	CD20+, EBER-, CD30-	NA	NA	NA
Aussedat *et al*., 2020 (Com-219) [16]	cHL-NS	PMBL	NA	CD30+, EBER-	CD20+, EBER-	NA	NA	NA
Aussedat *et al*., 2020 (Com-236) [16]	cHL-NS	DLBCL	NA	CD30+, EBER-	CD20+, EBER-, CD30-	NA	NA	NA
Aussedat *et al*., 2020 (Com-240) [16]	cHL-NS	PMBL	Separated (synchronous)	CD30+, EBER-	CD20+, EBER-	NA	NA	NA
Aussedat *et al*., 2020 (Com-251) [16]	cHL-LR	PMBL	NA	CD30+, EBER-	CD20+, EBER-, CD30-	NA	NA	NA
Aussedat *et al*., 2020 (Com-253) [16]	cHL-NS	GZL (group 2)	NA	CD30+, EBER-	CD20+, EBER-, CD30+	NA	NA	NA
Aussedat *et al*., 2020 (Com-312) [16]	cHL-NS	PMBL	NA	CD30+, EBER-	CD20+, EBER-	NA	NA	NA
Wilson *et al.,* 2014 [83]	cHL-NS	PMBL	NA	NA	NA	NA	NA	NA

Bellan *et al.,* 2002 [84]	cHL-NS	DLBCL	Separated	CD30+, CD15+, CD20-, CD79a-, CD3-, CD4-, CD5-, CD8-, EBER-, LMP1-, ALK1-, OCT2+	CD20+, CD79a+, CD30+ (weak), CD15-, EBER-, LMP1-, CD3-, CD4-, CD5-, CD8-	Common *Jh-Dh* rearrangement, different *Vh* segment rearrangement	Common *Jh-Dh* rearrangement, different *Vh* segment rearrangement	NA
Rosenquist *et al*., 2004 [13]	cHL-MC	DLBCL	NA	CD30+, CD15+ (focal), CD20-, EBER-, BOB1-, OCT2-	CD20+, CD30-, CD15-, EBER-, BOB1+, OCT2+	Common *IgK/IgH* rearrangement, with somatic hypermutations; Additional *IgK/IgH* rearrangement	Common *IgK/IgH* rearrangement, with somatic hypermutations (more important in DLBCL contingent)	NA
Wei *et al.,* 2004 [85]	cHL-NS	DLBCL	NA	CD30+, CD15+	CD20+, CD30-, CD15-, CD3-	NA	NA	NA
Traverse-Glehen *et al.,* 2005 (case 22) [86]	cHL-NS	PMBL	NA	CD30+ (weak), CD15+ (weak), CD20-, PAX5+, CD79a-, EBER-, BOB1-, OCT2-	CD20+, CD79a+, CD30+ (weak), CD15-, EBER-, PAX5+ (weak), BOB1+ (weak)	NA	NA	NA
Traverse-Glehen *et al.,* 2005 (case 23) [86]	cHL-NS	PMBL	Separated (synchronous)	CD30+ (weak), CD15+ (weak), CD20-, CD45-	CD20+, CD45+, MAL+, PAX5+, BOB1+, OCT2+	NA	NA	NA
Traverse-Glehen *et al.,* 2005 (case 24) [86]	cHL-NS	PMBL	NA	MAL+, PAX5+, EBER+, OCT2-, BOB1-	CD20+, CD45+, MAL+, CD30-, CD15-, EBER+, PAX5+, BOB1+, OCT2+	NA	NA	NA
Traverse-Glehen *et al.,* 2005 (case 25) [86]	cHL-NS	PMBL	NA	CD30+ (weak), CD15+ (weak), CD20-, CD45-, EBER-	CD20+, CD45+, CD30+ (weak), CD15-, EBER-	NA	NA	NA
Traverse-Glehen *et al.,* 2005 (case 26) [86]	cHL-NS	PMBL	NA	CD30+ (weak), CD15+ (weak), MAL-, CD20+ (weak), CD45-, PAX5+ (weak), CD79a+ (weak), EBER-, OCT2+ (weak), BOB1+ (weak)	CD20+, CD45+, MAL+, CD79a+, CD30-, CD15-, EBER-, BOB1+ (weak), OCT2+ (weak)	NA	NA	NA
Traverse-Glehen *et al.,* 2005 (case 27) [86]	cHL-NS	PMBL	Separated (synchronous)	CD30+ (weak), CD15+ (weak), CD20-, CD79a-	CD20+, CD30-, CD15-	NA	NA	NA
Quintanilla-Martinez *et al*., 2009 [87]	cHL-NS	PMBL	NA	CD30+, CD15+, EBER+	CD20+, MAL+, CD15-, EBER+	NA	NA	NA
Quintanilla-Martinez *et al*., 2009 [87]	cHL-NS	PMBL	NA	CD30+, CD15+, EBER+	CD20+, MAL+, CD15-, EBER+	NA	NA	NA
Quintanilla-Martinez *et al*., 2009 [87]	cHL-NS	PMBL	NA	CD30+, CD15+, EBER+	CD20+, MAL+, CD15-, EBER+	NA	NA	NA
Quintanilla-Martinez *et al*., 2009 [87]	cHL-NS	PMBL	NA	CD30+, CD15+, EBER+	CD20+, MAL+, CD15-, EBER+	NA	NA	NA
Oschlies *et al.,* 2011 [88]	cHL-NS	PMBL	NA	NA	NA	NA	NA	NA
Eberle *et al*., 2011 [89]	cHL-NS	PMBL	NA	NA	NA	NA	NA	NA
Yu *et al.,* 2011 [90]	cHL-NS	DLBCL	Separated	CD30+, CD15+, CD20-, PAX5-, CD79a-, CD10-, CD3-, CyclinD1-, BCL6-, EBER-, EMA-, MUM1+	CD20+, CD45+, CD79a+, BCL6-, CD10-, EBER-, CD3-, MUM1+, PAX5+	NA	NA	NA

Wang *et al.,* 2013 [91]	cHL-MC	DLBCL	Separated (synchronous)	CD30+, CD15+, CD20-, CD45-, PAX5+, CD79a-, CD10-, CD3-, EBER-, LMP1-, BOB1-, OCT2+, MUM1+	CD20+, CD45+, CD79a+, BCL6-, CD10-, CD30-, CD15-, EBER-, LMP1-, MUM1+, PAX+, Ki67 80%	Common *IgK* rearrangement	Common *IgK* rearrangement	NA
Khan *et al.*, 2017 [92]	cHL	PMBL	NA	CD30+, CD15+, PAX5+	CD20+, CD79a+, BCL2+ (weak), BCL6+ (weak), CD23+, CD10-, Ki67 70%	NA	NA	NA
Goyal *et al.*, 2016 [93]	cHL-MC	DLBCL	NA	CD30+, CD15-, CD20+ (weak), CD45-, PAX5+, CD10-, CD5-, CD3-, BCL6+ (weak), EBER-	CD20+, CD45+, BCL2+ (weak, focal), BCL6+, CD10-, CD30-, CD15-, EBER-, CD3-, CD5-, MUM1-, PAX5+	Common *Dh-Jh* rearrangement and additionnal *IgH* rearrangement	Common *Dh-Jh* rearrangement	No *BCL2, BCL6* or *MYC* translocation in both contingents
Khanna *et al.,* 2010 [94]	cHL-NS	DLBCL	Separated (synchronous)	EBER+	EBER+	Same clonal rearrangement (unspecified), different from the first DLBCL 3 years ago	Same clonal rearrangement (unspecified), different from the first DLBCL 3 years ago	NA
Auditeau *et al.,* 2018 [81]	cHL	DLBCL and MZL	NA	CD30+, CD15+, CD20-, CD79a-, ALK1-, CD5-, CD3-	CD20+ (cytometry), CD10+ (cytometry)	NA	NA	NA
Casey *et al.,* 1990 (case 1) [95]	cHL-NS	DLBCL	NA	CD15+, CD20-, CD45-, UCHL1-	CD20+, CD45+, UCHL1-, CD15-	NA	NA	NA
Casey *et al.,* 1990 (case 8) [95]	cHL-NS	DLBCL	Separated (synchronous)	CD15+, CD20-, CD45+, UCHL1-	CD20-, LN1/LN2+, CD45+, UCHL1-, CD15+ (focal)	NA	Clonal *IgH/IgK* rearrangement	NA
Guarner *et al.,* 1990 [96]	cHL-MC	DLBCL	Separated (synchronous)	EBER+	EBER+	NA	NA	NA
Huang *et al.,* 2006 [97]	cHL-NS	DLBCL	Separated with a transitional zone	CD30+, CD15+ (focal), CD20-, CD45+, PAX5-, CD79a-, CD10-, CD3-, BCL2+, BCL6-, EBER+, LMP1+, MUM1+	CD20+, CD45+, CD79a+, BCL2-, BCL6+, CD10-, CD3-, CD15-, EBER-, LMP1-, CD3-, MUM1-, PAX5+	Common *IgH* rearrangement	Common *IgH* rearrangement	NA
Wang *et al.*, 2020 [98]	cHL-MC	DLBCL	Separated (synchronous)	CD30+, CD15+ (focal), CD20+ (weak, focal), PAX5+, MYC+ (focal), CD3-, CD5-, CD10-, BCL2+ (weak), BCL6-, EBER+ (focal), LMP+, BOB1+, OCT2-, MUM1-	CD20+, MYC+, CD79a+, BCL2+ (focal), BCL6-, CD10-, CD30+, CD15-, EBER+ (focal), LMP1+ (focal), CD3-, CD5-, MUM1+, PAX5+, BOB1+, OCT2+, KI67 60%			NA
Kerl *et al.,* 1999 [99]	cHL-NS	High-grade B cell lymphoma	NA	CD30+, CD15+, CD20+	CD20+, CD30-, CD15-	Two different clonal rearrangements (unrelated)	Two different clonal rearrangements (unrelated)	NA
Perwein *et al.*, 2020 (case 4) [100]	cHL-NS	PMBL	Separated (synchronous)	CD30+, CD15+, CD20+ (focal), PAX5+, CD79a-,	CD20+, CD79a+, BCL6+, CD10+,	NA	NA	NA

				CD10-, BCL6-, EBER-	CD30+ (focal), CD15-, EBER-, PAX5+			
Perwein *et al*., 2020 (case 1) [100]	cHL-NS	DLBCL	NA	CD30+, CD15+ (focal), CD20+ (focal), PAX5+, CD79a-,	CD20+, CD79a-, CD30+, CD15-, PAX5+	NA	NA	NA
Miyagaki *et al*., 2019 [101]	cHL-NS	DLBCL	NA	CD30+, CD20-, CD4-, EBER+	CD20+, CD30-	NA	NA	NA
Paulli *et al.,* 1992 [102]	cHL-NS	DLBCL	NA	CD30+, CD15+, CD3-, CyclinD1-, UCHL1-, EMA-, CD45-	CD20+, CD45+, UCHL1-, CD30-, EMA-, CD15-, CD3-	NA	NA	NA
Hwang *et al.,* 2011 [103]	cHL-NS	DLBCL (EBV+ of the early)	NA	CD30+, CD15+, EBER+	CD20+, CD30-, EBER+	No clonal *IgH* or *IgK* rearrangement	No clonal *IgH* or *IgK* rearrangement	NA
Hell *et al*., 1995 (case 3) [104]	cHL-NS	DLBCL	NA	CD30+, CD15+, CD20+, CD3-	CD20+, CD30-, CD15-, CD3-	NA	NA	NA
Hell *et al*., 1995 (case 6) [104]	cHL-NS	DLBCL	NA	CD30+, CD15+, CD20-, CD3-	CD20+, CD30-, CD15-, CD3-	NA	NA	NA
Soliman *et al.,* 2016 [105]	cHL-MC	Burkitt lymphoma/leukemia	Separated (synchronous)	CD30+, CD15+, CD20-, CD45-, CD3-, LMP1+	CD20+, MYC+, CD79a+, BCL2-, BCL6+, CD10+, EBER+, CD3-, Ki67 95%	No *IgH* rearrangement	*IgH* rearrangement	*IgH/MYC* translocation in the Burkitt contingent only; Cytogenetic analysis : t(8;14)(q24;q32)
Kim *et al.,* 1977 (case 18) [1]	cHL-NS	DLBCL	Separated (synchronous) and composite	NA	NA	NA	NA	NA
Jaffe *et al.,* 1992 (case 3) [58]	cHL	DLBCL	NA	CD15+, CD20-	NA	NA	NA	NA
Jaffe *et al.,* 1992 (case 6) [58]	cHL	DLBCL	NA	NA	NA	NA	NA	NA
Jaffe *et al.,* 1992 (case 7) [58]	cHL	DLBCL	NA	CD15-, CD20-	NA	NA	NA	NA
Jaffe *et al.,* 1992 (case 8) [58]	cHL	DLBCL	NA	CD15+, CD20-	NA	NA	NA	NA
Jaffe *et al.,* 1992 (case 9) [58]	cHL	DLBCL	NA	NA	NA	NA	NA	NA
Jaffe *et al.,* 1992 (case 10) [58]	cHL	DLBCL (intermediate cells)	NA	CD15-, CD20-	NA	NA	NA	NA
Jaffe *et al.,* 1992 (case 11) [58]	cHL	DLBCL	NA	NA	NA	NA	NA	NA
Jaffe *et al.,* 1992 (case 12) [58]	cHL	DLBCL	NA	CD15+, CD20+	NA	NA	NA	NA
Jaffe *et al.,* 1992 (case 14) [58]	cHL	DLBCL	Separated (synchronous)	CD15+, CD20-	NA	NA	NA	NA
Jaffe *et al.,* 1992 (case 15) [58]	cHL	DLBCL	Separated (synchronous)	CD15+, CD20-	NA	NA	NA	NA

cHL: classical Hodgkin lymphoma; DLBCL: Diffuse large B cell lymphoma; EBER: Epstein-Barr virus-encoded small RNA *in situ* hybridization; EBV: Epstein-Barr virus; GZL: Grey zone lymphoma; LR: Lymphocyte rich; MC: Mixed cellularity; MZL: Marginal zone lymphoma; NA: Not available; NS: Nodular sclerosis; PMBL: Primary mediastinal B cell lymphoma; +: positive; -: negative.

*Case from the study of Kigma et al., 1994 [65] in the synchronous/simultaneous lymphomas table. **Major/predominant contingent when indicated.

Supplementary Table S10: clinical and laboratory data of cHL/NLPHL composite lymphomas.

References	Age (years)	Sex	History of lymphoma	Location	Tumor size (cm)	B symptoms	Ann Arbor stage	Other symptoms or tumor mass/LN	Laboratory data	Extension assessment	Treatment	Outcome	Follow-up duration (months)	Time of relapse / DOD (months)
Song *et al.,* 2011 [106]	24	M	Familly history of cHL (father and two paternal cousins) and myeloid leukemia (grandfather)	Post-auricular parotid gland and thymic mass	3 and 2	Yes	VI B	No	NA	PET-scan : hypermetabolism of thymic mass; BM-.	NA	NA	NA	NA
Szczepanowski *et al.,* 2013 [107]	48	M	No	Cervical, supraclavicular, axillary LN	NA	No	III A	No	NA	CT-scan: involvement of spleen and liver; BM-	ABVD x4 + RT, and then BEACOPP x4	Partial remission to the first chemotherapy and then complete remission after 10 months	12	NA
Gelb *et al.,* 1993 (case 5) [108]	22	M	NA	Submandibular LN	NA	No	I A	No	NA	NA	RT	Complete remission	NA	NA

BM+/-: Involvement or not on bone marrow biopsy; cHL: classical Hodgkin lymphoma; CT-scan: computed tomography-scan; LN: Lymph node(s); M: Male; NA: Not available; NLPHL: Nodular lymphocyte predominant Hodgkin lymphoma; PET-scan: Positron emission tomography-scan; RT: Radiotherapy. For chemotherapy abbreviations, please see **Supplementary Text S1**.

Supplementary Table S11: histopathological and molecular data of cHL/NLPHL composite lymphomas.

References	cHL type	NLPHL	Separated / mixed contingents	Immunophenotype cHL	Immunophenotype MZL	Clonal B rearrangements (cHL)	Clonal B rearrangements (NLPHL)
Song *et al.*, 2011 [106]	CHL-NS	NLPHL	Separated (synchronous)	CD30+, CD15+, CD20-, PAX5+, IgD-, p53+, BCL6-, EBER-, EMA-, OCT2-	CD20+, BCL6+, p53+, CD30+ (weak), CD15-, EBER-, EMA-, PAX5+, OCT2+, IgD-	Polyclonal *IgH* rearrangements, common *IgK* rearrangement	Polyclonal *IgH* rearrangements, common *IgK* rearrangement
Szczepanowski *et al.,* 2013 [107]	CHL-MC	NLPHL	Separated	CD30+, CD15+, CD20+ (focal/weak), PAX5+, CD79a-, CD45-BCL6-, EBV+, LMP1+, BOB1-, OCT2+, MUM1+	CD20+, CD45+, CD79a+, BCL6+, CD30-, CD15-, EBER-, LMP1-, MUM1+, PAX5+, BOB1+, OCT2+	Common *IgH* rearrangement Common somatic hypermutations of *V* gene	Common *IgH* rearrangement and second non shared *IgH* allel Common somatic hypermutations of *V* gene
Gelb *et al.*, 1993 (case 5) [108]	CHL-MC*	NLPHL	Separated	CD30+, CD15+	CD45-, CD30-, CD15-	NA	NA

cHL: classical Hodgkin lymphoma; EBER: Epstein-Barr virus-encoded small RNA *in situ* hybridization; MC: Mixed cellularity; NA: Not available; NLPHL: Nodular lymphocyte predominant Hodgkin lymphoma; NS: Nodular sclerosis; +: positive; -: negative.

*Predominant contingent.

Supplementary Table S12: Clinical and laboratory data of cHL/T-cell composite lymphomas.

References	Age (years)	Sex	History of lymphoma	Location	Tumor size (cm)	B symptoms	Ann Arbor stage	Other symptoms or tumor mass/LN	Laboratory data	Extension assessment	Treatment	Outcome	Follow-up duration (months)	Time of relapse / DOD (months)
Ichikawa *et al.*, 2017 (case 1) [109]	76	F	No	NA	NA	NA	IV A	NA	NA	NA	NA	NA	NA	NA
Ichikawa *et al.*, 2017 (case 2) [109]	19	M	HL (2010)	NA	NA	NA	II A	NA	NA	NA	ICE, LEED, Auto-PBSCT	Complete remission, alive	48	NA
Ichikawa *et al.*, 2017 (case 3) [109]	74	M	No	NA	NA	NA	III A	NA	NA	NA	ABVD	Complete remission, alive	36	NA
Ichikawa *et al.*, 2017 (case 4) [109]	43	M	No	NA	NA	NA	I A	NA	NA	NA	ABVD	Complete remission, alive	24	NA
Ichikawa *et al.*, 2017 (case 5) [109]	57	F	T-cell prolymphocytic leukemia (2002)	NA	NA	NA	IV A	NA	Atypical lymphocytes in peripheral blood	NA	ABVD	Partial remission, dead at Sep/2015	NA	NA
Gualco *et al.*, 2009 [110]	55	F	No	Cervical LN and mediastinal	NA	Yes	II B	NA	Anemia, LDH and β2-microglobulin increase	CT-scan: mediastinal lymphadenopathy	NA	NA	NA	NA
Sanchez *et al.*, 2006 [11]	65	M	No	Cervical, axillary, right groin, iliac, and splenomegaly and liver involvement	2,1	Yes	NA	Dysuria	Normal blood cell count and chemistry panel	CT-scan: extensive lymphadenopathy throughout the abdomen and pelvis, splenomegaly	Cyclophosphamide, prednisone and then C-MOPP	Dead of sepsis and hepatic encephalopathy after 3 weeks	0.75	0.75
Brown *et al.*, 2004 [112]	60	M	No	Cervical LN	NA	No	IV A	Fever and night sweats	NA	BM+: PTCL (20-30% involved)	CHOP x4 at first, then ESHAP, and then autologous-PBSCT after relapse	NA	NA	NA
Wlodarska *et al.*, 1993 [113]	54	M	HL 3 years earlier	Cervical, axillary, mediastinal, retroperitoneal LN	NA	Yes	NA	Arthritis	NA	BM-	NA	NA	NA	NA
Gui *et al.*, 2020 [114]	44	M	No	Bilateral axillary, cervical, inguinal, mediastinal, parailliac, retroperitoneal LN	4	No	IV A	NA	LDH and β2-microglobulin in normal range	PET-scan: bilateral axillary, cervical, inguinal, mediastinal and parailliac, retroperitoneal LN BM+: T-cell lymphoma (3,5%)	ABVD x1, chidamide, followed by GDP x1	Partial remission	NA	NA
Demurtas *et al.*, 2011 (case 17) [60]	84	M	No	Axillary LN	NA	NA	NA	NA	NA	NA	NA	NA	Loss to follow-up	Loss to follow-up

BM+/-: Involvement or not on bone marrow biopsy; cHL: classical Hodgkin lymphoma; CT-scan: computed tomography-scan; F: Female; LDH: Lactate dehydrogenase; LN: Lymph node(s); M: Male; MF: Mycosis fungoid; NA: Not available; PET-scan: Positron emission tomography-scan; PTCL: Peripheral T-cell lymphoma; RT: Radiotherapy. For chemotherapy abbreviations, please see **Supplementary Text S1**.

Supplementary Table S13: histopathological and molecular data of cHL/T-cell composite lymphomas.

References	cHL type	T-cell lymphoma type	Separated / mixed contingents	Immunophenotype cHL	Immunophenotype T-cell lymphoma	Clonal B rearrangements (cHL)	Clonal T rearrangements (T-cell lymphoma)	Other molecular alterations
Ichikawa *et al.*, 2017 (case 1) [109]	cHL-MC	PTCL	NA	CD30+, CD15-, CD20-, PAX5+, EBER-	CD3+, CD4-, CD8+, TIA1+, Granzyme B-, EBER-	NA	NA	On whole tissue sample: clonal rearrangements of *IgH* and TCRγ
Ichikawa *et al.*, 2017 (case 2) [109]	cHL-MC	PTCL	NA	CD30+, CD15-, CD20+, PAX5+, EBER-	CD3+, CD4-, CD8+, TIA1+, Granzyme B-, FOXP3-, EBER-	NA	NA	On whole tissue sample: clonal rearrangements of *IgH* and TCRγ
Ichikawa *et a*l., 2017 (case 3) [109]	cHL-MC	PTCL	NA	CD30+, CD15+, CD20-, PAX5+, EBER-	CD3+, CD4+, CD8-, TIA1-, Granzyme B-, FOXP3+, EBER-	NA	NA	On whole tissue sample: clonal rearrangements of *IgH* and TCRγ
Ichikawa *et al.*, 2017 (case 4) [109]	cHL-MC	T-cell prolymphocytic leukemia	NA	CD30+, CD15+, CD20-, PAX5+, EBER-	CD3+, CD4-, CD8+, TIA1+, Granzyme B-, EBER-	NA	NA	On whole tissue sample: clonal rearrangements of *IgH* and TCRγ
Ichikawa *et al.*, 2017 (case 5) [109]	cHL-MC	T-cell prolymphocytic leukemia	NA	CD30+, CD15+, CD20-, PAX5+, EBER-	CD3+, CD4-, CD8+, TIA1+, Granzyme B-, EBER-	NA	NA	On whole tissue sample: clonal rearrangements of *IgH* and TCRγ
Gualco *et al.*, 2009 [110]	cHL	PTCL	NA	CD30+, CD15+, CD20-, EBER+, LMP1+, CD3-, CD45-	CD30-, CD15-, CD3+, CD4+, CD8-, CD56-, Ki67: 80%	Clonal *IgH* rearrangement	Clonal TCRγ and β rearrangement	NA
Sanchez *et al.*, 2006 [111]	cHL	PTCL	NA	CD30+, CD15+, CD20-, EBER+, CD45-, ALK1-	CD3+, CD5-	NA	NA	On whole tissue sample: TCRγ rearrangement : same rearrangements on both sites with both composite lymphomas No *IgH* rearrangement
Brown *et al.*, 2004 [112]	cHL-NS	PTCL	Separated (synchronous)	CD30+, CD15+, CD20-, EBER-, CD3-, CD45-	CD30-, CD15-, CD3+, CD5+, CD7-, CD45+, CD10-	NA	NA	Similar clonal TCRγ rearrangement in both site involved by T-cell lymphoma
Wlodarska *et al.*, 1993 [113]	cHL	PTCL	Mixed	CD30+, CD15+, CD20+, CD3-, CD4-, CD5-, CD8-	CD30-, CD15-, CD3+, CD4+, CD5+, CD8-, CD45+	NA	NA	On whole tissue sample: TCRγ and β rearrangements
Gui *et al.*, 2020 [114]	cHL	Cytotoxic CD8+ T-cell lymphoma	NA	CD30+, CD15+, PAX5+, MUM1+, EBER+, Ki67: 60%	CD30-, CD20-, CD2+, CD3+, CD4+ (weak), CD5+, CD7+, CD8+, TIA1+, Granzyme B-, , CD10-, CXCL13-, PD1+, CD56-, Ki67: 40%	NA	NA	On whole tissue sample: TCRβ and δ rearrangements
Demurtas *et al.*, 2011 (case 17) [60]	cHL	PTCL	NA	NA	CD2+, CD3+, CD5-, CD7-, CD8+	No clonal *IgH* rearrangement	Clonal TCRγ rearrangement	NA

cHL: classical Hodgkin lymphoma; MC: Mixed cellularity; NS: Nodular sclerosis; EBER: Epstein-Barr virus-encoded small RNA *in situ* hybridization; NA: Not available; PTCL: Peripheral T cell lymphoma; +: positive; -: negative.
